# Recapitulative haematopoietic development of human pluripotent stem cells in the absence of exogenous haematopoietic cytokines

**DOI:** 10.1111/jcmm.16826

**Published:** 2021-08-02

**Authors:** Elena S. Philonenko, Ying Tan, Cuihua Wang, Baoyun Zhang, Zahir Shah, Jianguang Zhang, Hanif Ullah, Sergei L. Kiselev, Maria A. Lagarkova, Dandan Li, Yong Dai, Igor M. Samokhvalov

**Affiliations:** ^1^ CAS Key Laboratory of Regenerative Biology Guangdong Provincial Key Laboratory of Stem Cells and Regenerative Medicine Guangzhou Institutes of Biomedicine and Health Chinese Academy of Sciences Guangzhou China; ^2^ Vavilov Institute of General Genetics Russian Academy of Sciences Moscow Russia; ^3^ University of Chinese Academy of Sciences Beijing China; ^4^ Federal Research and Clinical Center of Physical‐Chemical Medicine of Federal Medical Biological Agency Moscow Russia; ^5^ Clinical Medical Research Center Guangdong Provincial Engineering Research Center of Autoimmune Diseases and Precision Medicine Shenzhen People’s Hospital The First Affiliated Hospital of Southern University of Science and Technology The Second Clinical Medical College of Jinan University Shenzhen China

**Keywords:** haematopoiesis, haematopoietic stem cells, human haematopoietic development, human pluripotent stem cells, inflammation

## Abstract

To improve the recapitulative quality of human pluripotent stem cell (hPSC) differentiation, we removed exogenous haematopoietic cytokines from the defined differentiation system. Here, we show that endogenous stimuli and VEGF are sufficient to induce robust hPSC‐derived haematopoiesis, intensive generation of haematopoietic progenitors, maturation of blood cells and the emergence of definitive precursor cells including those that phenotypically identical to early human embryonic haematopoietic stem cells (HSCs). Moreover, the cytokine‐free system produces significantly higher numbers of haematopoietic progenitors compared to the published protocols. The removal of cytokines revealed a broad developmental potential of the early blood cells, stabilized the hPSC‐derived definitive precursors and led to spontaneous activation of inflammatory signalling. Our cytokine‐free protocol is simple, efficient, reproducible and applicable for embryonic stem cells (ESCs) and induced PSCs. The spectrum of recapitulative features of the novel protocol makes the cytokine‐free differentiation a preferred model for studying the early human haematopoietic development.

## 1 INTRODUCTION

In vitro differentiation of human pluripotent stem cells (hPSCs) has been widely used for the generation of clinically relevant cell populations and as a model for studying the early human development.^1^, ^2^, ^3^ Selecting culture conditions for closer mimicking of haematopoietic development in the conceptus can improve functional characteristics of hPSC‐derived haematopoietic cells. It can also create a better model for studying the mechanisms of the early human haematopoietic development.

The early haematopoiesis in mammals is essentially planar,^4^, ^5^ which makes the two‐dimensional hPSC differentiation more recapitulative compared to 3D approaches. Stromal cells that have been extensively used for hPSC differentiation are derived from relatively late embryonic or foetal haematopoietic sites^6^, ^7^, ^8^, ^9^ and therefore are unlikely to create a recapitulative environment for the initiation of haematopoietic development. In this regard, the most promising strategy is a planar differentiation system in defined culture conditions on surfaces covered by extracellular matrix proteins.^10^, ^11^ The matrix proteins seem to recapitulate the extracellular part of the transitory Heuser's membrane, which is adjacent to the primary extraembryonic mesoderm in the early human conceptus. Furthermore, the planar differentiation of hPSCs on extracellular matrix in a defined medium is preferred due to its simplicity, higher consistency and safety.^1^, ^11^

The common feature of all protocols for the hPSC‐derived haematopoiesis is the stimulation of blood cell generation by a cocktail of exogenous cytokines.^12^ The haematopoietic cytokines are generally used at high, non‐physiological concentrations. Given that cytokines are known to have an instructive role in lineage specification,^13^, ^14^ their usage in hPSC differentiation can distort the haematopoietic development and prevent faithful recapitulation of the in vivo events.

An attempt to generate blood cells from mouse ESCs in a defined medium without exogenous growth factors and cytokines failed but a combination of BMP4 and VEGF was capable to induce haematopoiesis in the absence of cytokines.^15^ The cytokine‐free embryoid body (EB)–mediated differentiation induced the emergence of cells expressing the erythroid marker Ter119 and clonogenic haematopoietic progenitors. However, it was not clear whether these progenitors emerged in the primary differentiation or during the cytokine‐supplemented assay medium. Similar results were obtained using a more complicated mixture of growth factors to induce haematopoietic differentiation of mESCs.^16^ The haematopoietic differentiation beyond the commitment stage has not been studied, and it remained unclear whether sustained haematopoietic development was possible in the absence of exogenous cytokines.

In this study, we have shown that planar differentiation of hPSCs in defined conditions and the absence of exogenous haematopoietic cytokines creates a self‐regulatory system of haematopoietic development and initiates sustained haematopoiesis accompanied by activation of inflammatory signalling. We characterized the novel differentiation system in detail and demonstrated that it is highly efficient, consistent and closely recapitulates the early stages of human haematopoietic development.

## 2 MATERIALS AND METHODS

### 2.1 Culturing hPSCs

All hESC (H1/WA01, HN14, MEL1) and hiPSC (iPS12, iPSS1 and iPSS9)^17^ lines were maintained in the undifferentiated state on Matrigel‐coated plates (Corning Matrigel, Cat. No. 354230) in mTeSR1 medium (STEMCELL Technologies, Vancouver, Canada). Before transfection and haematopoietic differentiation, hPSCs were subjected to at least three short passages (2–3 days) at a seeding density of 4–6 × 10^6^ cells per one well of a standard 6‐well culture plate.

### 2.2 Haematopoietic differentiation of hPSCs

For EB formation, usually 1 × 10^6^ single cells were spun at 100 × g for 4 min into AggreWell^TM^400 (STEMCELL Technologies) in mTESR1 medium containing 1 μM Thiazovivin and incubated for 20–24 h at 37°C and 5% CO_2_. The newly formed EBs were induced to attach to Corning^®^ mCollagen IV (Corning Life Sciences)–coated surfaces under normal gravity in mTeSR1 medium supplemented with 2–4 ng/ml hBMP4 (PeproTech, Rocky Hill, NJ), 50 ng/ml hVEGF_165_ (PeproTech) and 10 μM Thiazovivin. After 48 h of the EB attachment, the medium was replaced with pre‐warmed 2 ml/well of the Haematopoietic Medium (StemLine^TM^ II HSC expansion medium (Sigma‐Aldrich, St. Louis, MO), 1 × GlutaMax, 1 × 2‐Mercaptoethanol, 1 × NEAA, and 50 ng/ml hVEGF_165_).

### 2.3 Flow cytometry and cell sorting

For flow cytometry and cell sorting, we used anti‐human monoclonal antibodies from Becton Dickinson (BD Life Sciences, Franklin Lakes). All sorting procedures were performed on either BD FACSAria II or Beckman Coulter MoFlo Astrios machines. The flow cytometry analyses were done on BD C6 Accuri and BD LSRFortessa. The data were analysed with FlowJo V10 (FlowJo LLC, BD) and BD C6 Accuri software.

### 2.4 Haematopoietic progenitor assay

Haematopoietic progenitor assay was performed in the serum‐free methylcellulose medium SF H4436 (STEMCELL Technologies) according to the manufacturer's recommendations in duplicates for at least two different cell densities of each input cell population ranging from 5 × 10^3^ to 5 × 10^4^ cells per 1.5 ml of the medium per one 35 mm Petri dish. The colonies were grown for 16–18 days at 37°C in the humidified atmosphere containing 5% CO_2_.

### 2.5 Lymphoid differentiation

A total of 2–5 × 10^5^ cells of MACS‐enriched hESC‐derived cell fractions (CD45^+^CD34^low/−^ and CD34^+^CD45^low/−^) were added to the individual well of a 6‐well plate containing OP9‐DL4 cells and cultured in the OP9 Differentiation Medium (α‐MEM, 20% FBS and 50 µM 2‐Mercaptoethanol) supplemented with rhSCF (5 ng/ml), rhIL‐7 (5 ng/ml) and rhFLT3‐L (5 ng/ml) (all from PeproTech). Every 7 days, differentiating cells were replated onto the fresh OP‐DL4 stroma.

### 2.6 Transcriptional profiling by RNA sequencing

H1 hESCs at various stages of the haematopoietic differentiation were used for RNA sequencing as follows: unsorted total live cells for undifferentiated hESCs and day 0, 2, 4 differentiated cells; sorted CD43^+^ and CD43^−^ cell populations at days 6, 9, 12; CD43^−^CD45^−^ and CD43^+^/CD45^+^ populations at day 16 of differentiation. Three independent biological replicates were prepared for each cell population at each differentiation stage totalling 34 samples. RNA samples were sequenced at RiboBio and Annoroad Gene Technology.

Additional information on materials and methods is provided in the Supplemental Materials and Methods.

## 3 RESULTS

### 3.1 Design of the cytokine‐free haematopoietic differentiation of hPSCs in defined conditions

**FIGURE 1:**
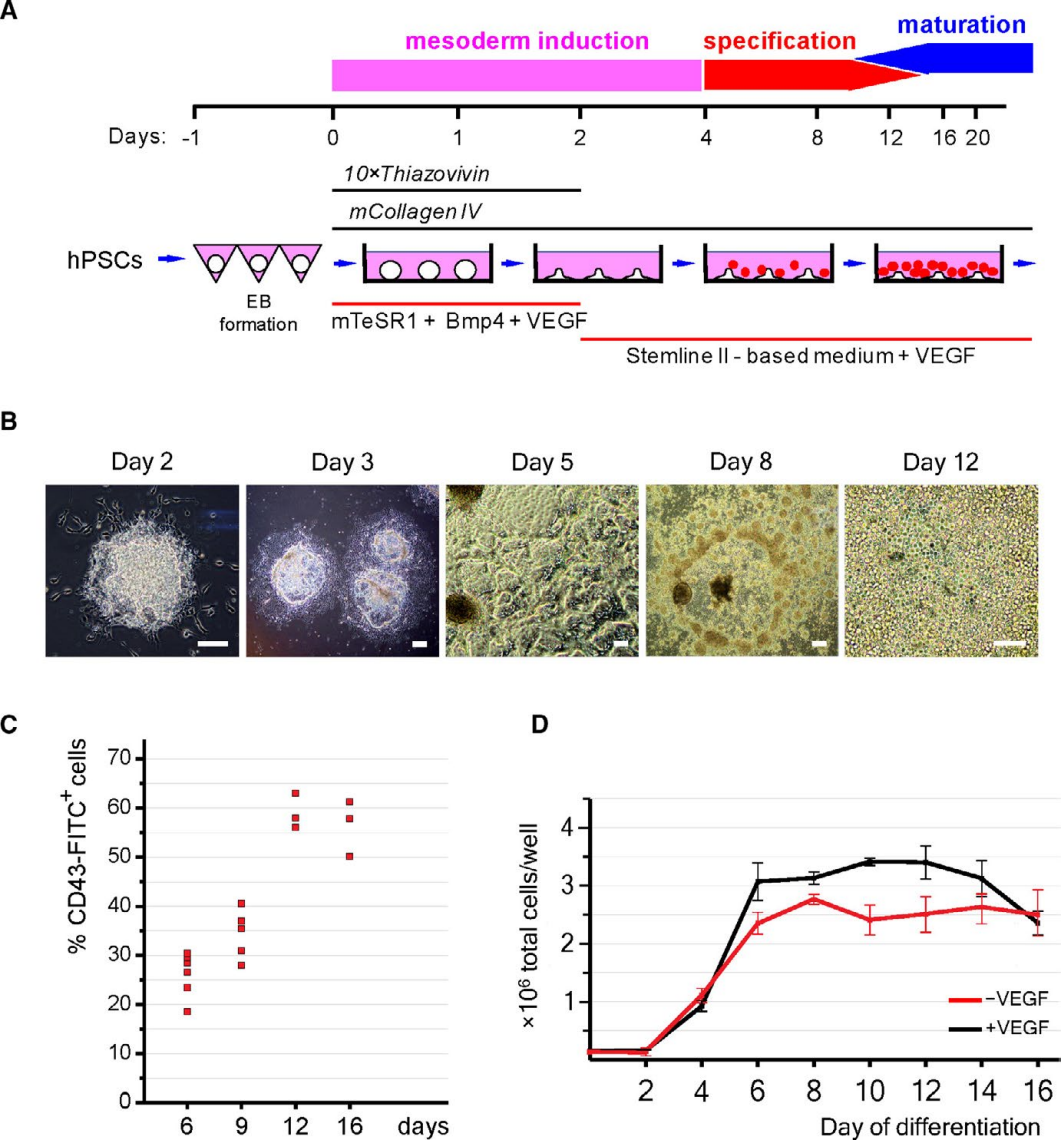
Planar cytokine‐free haematopoietic differentiation of hPSCs in defined culture conditions is highly proliferative and reproducible. (A) Scheme of the planar hPSC differentiation in the absence of haematopoietic cytokines. (B) Cell morphology at indicated days of the differentiation. Scale bar–100 μm. (C) Differentiation of H1 hESCs reproducibly generates CD43^+^ blood cells at the key stages of the differentiation. (D) Cell number dynamics in the H1 hESC differentiating cultures in the presence and the absence of VEGF. Measurements are shown for one well of a standard 6‐well cell culture plate. Data are mean ± SD, *n* = 3

To initiate mesodermal and haematoendothelial development, hPSCs were replated onto collagen IV–coated surfaces (Figure 1A). To this end, we first generated uniformly sized EBs and then subjected them to the unforced attachment to collagen IV under normal gravity. To stimulate the attachment of 24‐hour‐old EBs, the medium was supplemented with 10 µM (10×) Thiazovivin. The attachment led to a fast circumferential expansion of EB‐derived cells over the culture surface creating flattened EB‐centred colonies of differentiating cells (Figure 1B). The transition from a clump of pluripotent cells to a sheet of differentiating cells mimics the essential planar expansion of the hypoblast in the post‐implantation embryos.

In our differentiation system, collagen IV, tenascin‐C and fibronectin displayed similar efficiency in support of the hPSC‐haematopoiesis (Figure S1A). The minimal combination of VEGF and BMP4 was sufficient to induce potent mesodermal specification during the first 48 h of the EB attachment. BMP4 was used at a low concentration (2–4 ng/ml) to minimize the generation of trophectoderm cells.^18^ After the attachment, the only exogenous growth factor used for human blood and progenitor cell generation was VEGF. This endothelial factor supported the emergence and segregation of the three major mesodermal cell lineages: CD31^low/+^CD43^+^ early blood cells, CD31^+^CD43^−^CD146^+^ endothelial cells and CD31^–^CD43^–^CD146^low/+^ mesenchymal cells (Figure S1B) and induced a strong expression of CD45 within the CD43^+^ cell population (Figure S1C). Addition of FGF2 (5–20 ng/ml) was found to be inhibitory for the cytokine‐free differentiation (Figure S1D).

The protocol has only a couple of variables that can influence the efficiency of hPSC differentiation. First, high densities of the EB plating strongly suppressed haematopoietic differentiation suggesting paracrine inhibition of hPSC‐haematopoiesis. Second, even though all tested hESC and hiPSC lines were able to initiate haematopoiesis and generate clonogenic progenitors, the efficiency of the haematopoietic differentiation varied significantly (Figures S2A,B). The hESCs showed less fluctuating haematopoietic potential compared to the hiPSCs. Notwithstanding these variations and providing that all manipulations and conditions are standardized, the protocol is remarkably reproducible based on the measurement of haematopoietic marker expression (Figure 1C).

### 3.2 Characterization of the cytokine‐free differentiation

Despite the absence of exogenous haematopoietic cytokines, the haematopoietic differentiation of hPSCs was accompanied by intensive cell proliferation (Figure 1D), so that by day 12 non‐adherent haematopoietic cells completely covered differentiating cultures (Figure 1B). If VEGF is removed after day 2, the early haematopoiesis is strongly suppressed but the culture continues to proliferate in the absence of any exogenous stimulatory molecules eventually giving rise to large populations of myeloid cells.^19^ The ability to proliferate without VEGF (Figure 1D) suggests that in such conditions the early VEGF‐dependent blood cells are replaced by VEGF‐independent cell lineages.

**FIGURE 2:**
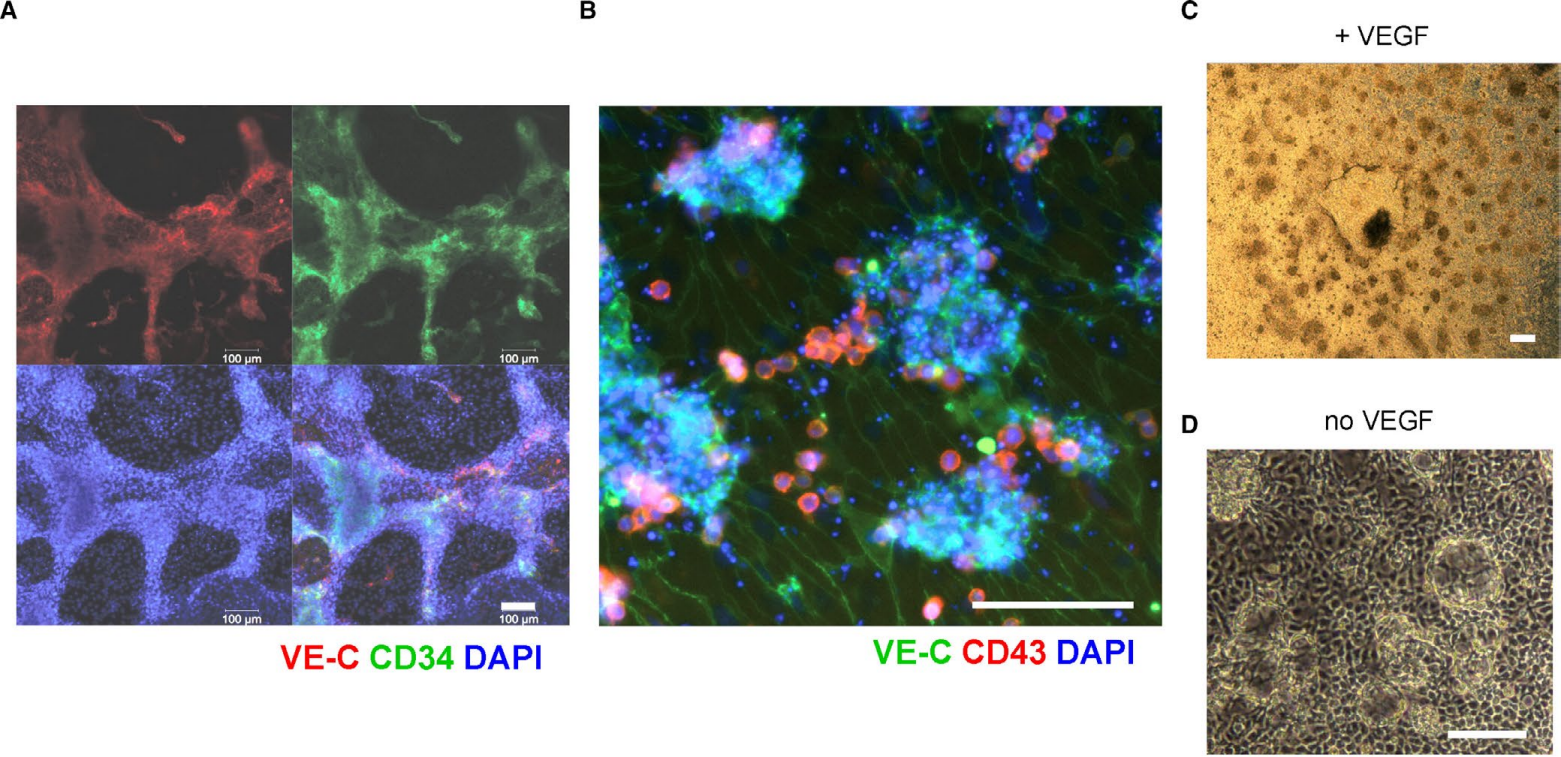
Spontaneous formation of the yolk sac–type angioblastic cords and blood islands in the cytokine‐free system. (A) Immunocytofluorescent staining of VE‐CADHERIN^+^/CD34^+^ endothelial/angioblastic cords on day 5 of the H1 hESC differentiation. (B) The staining reveals that day 8 in vitro blood islands (IBIs) generate the earliest CD43^+^ blood cells in a process similar to EHT. (C) If VEGF is constantly present in the differentiation medium, each EB attachment site is surrounded by its own set of IBIs of variable sizes. (D) If VEGF is removed after the EB attachment, IBIs failed to form turning into ring structures that look like the IBI rudiments. (C) and (D) pictures are taken on day 6 of H1 hESC differentiation. Scale bars in all panels–100 μm

The EB attachment stimulated a strong planar outgrowth of mesodermal cells followed by the formation of VE‐CADHERIN^+^ endothelial cords and a monolayer of endothelial cells (Figure 2A,B). The expansion of the endothelial cords led to their spontaneous dissociation into characteristic transitory VE‐CADHERIN^+^ cell aggregates sitting on top of the endothelial sheet (Figure 2B). Practically each EB attachment site was surrounded by its own set of the aggregates (Figure 2C). These aggregates were responsible for the emergence of the first non‐adherent CD43^+^ blood cells, and by analogy with similar yolk sac aggregates, we termed them ‘in vitro blood islands’ (IBIs). After apparent downregulation of VE‐CADHERIN in the peripheral cells, single or clumped VE‐CADHERIN^–^CD43^+^ haematopoietic cells emerged on IBI fringes (Figure 2B). The loss of VE‐CADHERIN was followed by dissociation of the first blood cells from IBIs in a process that was reminiscent of the endothelial‐to‐haematopoietic transition (EHT). The IBIs were visible for 3–4 days and vanished when their cellular contents turned into non‐adherent blood cells. If VEGF was removed after the EB attachment, the IBIs failed to form, leaving corresponding rudimentary non‐haematopoietic structures (Figure 2D). Thus, the single growth factor is sufficient to induce hPSC‐derived endothelial cells to initiate haematopoiesis. The emergence of the transitory endothelial cords and EHT‐competent IBIs strongly suggests that our differentiation system closely recapitulates the early haematopoietic development in the mammalian conceptus.

**FIGURE 3:**
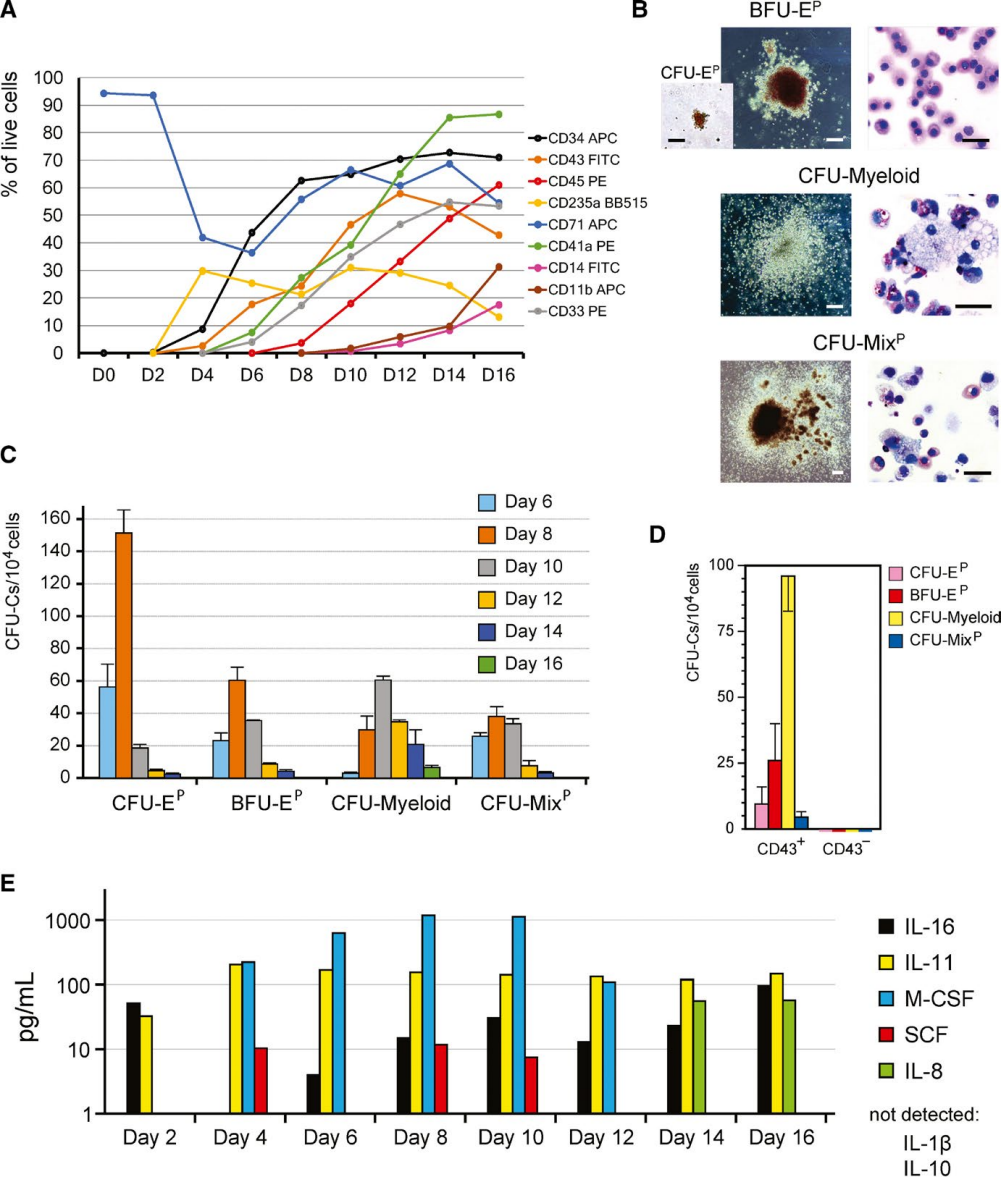
Robust hPSC‐derived haematopoiesis does not require haematopoietic cytokines. (A) Representative dynamics of marker expression during haematopoietic differentiation of H1 hESCs. (B) Types of primitive clonogenic progenitors generated by cytokine‐free differentiation of hPSCs. Scale bars–colony pictures, 100 μm; May‐Grünwald cell staining–20 μm. (C) Dynamics of the haematopoietic progenitor generation during H1 differentiation. Data are mean ± SD, *n* = 4. (D) The primitive clonogenic progenitors are CD43‐positive. The data (mean ± SD, *n* = 4) for IPS12 hiPSC line are shown. (E) Time‐course ELISA quantitation of the endogenous haematopoietic cytokines synthesized by H1 cells

The expression dynamics of haematopoietic markers indicated an early start of haematopoiesis (Figure 3A). Unattached EBs at day 0 expressed CD71, the transferrin receptor that apart from being expressed in erythroid cells is also a cell surface marker of cell proliferation.^20^ A significant drop in the CD71 expression between day 2 and day 4 suggests a substantial decline in cell proliferation during the migration of cells escaping from the attached EBs. CD235a, a marker of human erythroid cells, and CD34, a marker of haematopoietic progenitors and endothelial cells, began their expression already at day 4 of the differentiation. CD235a along with CD41a was shown to mark the hPSC‐derived primitive haematopoiesis..^21^, ^22^ First CD45‐positive cells were noticed on days 8–9, while reliable expression of CD11b and CD14 was observed only at day 16, which suggests the maturation of monocyte/macrophage and granulocyte lineages. The staged expression of the cell surface haematopoietic markers reflects the developmental dynamics of hPSC‐derived haematopoietic cells.

Next, we examined the generation of clonogenic haematopoietic progenitors in our differentiation system (Figure 3B). Primitive erythroid progenitors emerged already on day 6 of the differentiation. These progenitors generated colonies of primitive erythroblasts expressing high levels of the embryonic haemoglobin ε.^19^ The largest numbers of these CFU‐E^P^ (colony forming units–erythroid, primitive) and more proliferative BFU‐E^P^ (burst forming units–erythroid, primitive) progenitors were detected on day 8; these primitive erythroid progenitors were still detectable at day 10 and gradually disappeared afterwards. The wave of granulocyte‐macrophage progenitors, CFU‐Myeloid, emerges 2 days later and continues long into day 16. The dynamics of BFU‐E^P^ and multilineage CFU‐mix^P^ progenitors were similar; the mixed colonies contained primitive megakaryocytes, macrophages and granulocytes in addition to primitive erythroblasts (Figure 3C). All haematopoietic progenitors expressed CD43 (Figure 3D), which at the early stages of hPSC differentiation marks the primitive haematopoiesis.^21^, ^22^

The removal of exogenous cytokines has been considered as a measure interfering with the loss of haematopoietic progenitors, whose numbers nevertheless dropped at the later stages of differentiation. This observation is consistent with the inherent transience of the primitive haematopoietic progenitor generation. A possible reason for the progenitor decline is the endogenous generation of haematopoietic cytokines that may induce overt differentiation of the emerging haematopoietic progenitors. Our analysis of the haematopoietic cytokine synthesis confirmed the presence of relatively high levels of endogenous CSF1 and IL11 (Figure 3E). Both cytokines are strong modulators of myelopoiesis.^23^, ^24^ Nevertheless, our cytokine‐free differentiation system generated significantly, up to one order of magnitude, higher numbers of clonogenic progenitors compared to cytokine‐supported differentiation of the same hPSC line using EBs, OP9 stroma or extracellular matrix proteins.^6^, ^11^, ^25^, ^26^ This observation strongly suggests that the omission of cytokines reduces the loss of hPSC‐derived clonogenic haematopoietic progenitors.

We also investigated whether pharmacological manipulations can regulate the haematopoietic development in our system. Activin A strongly promoted, while SB‐431542 (SB), the inhibitor Activin/Nodal signalling, drastically suppressed the primitive haematopoiesis.^19^ CHIR99021 (CHIR), a Wnt agonist, also had a profound negative effect on the emergence of primitive haematopoietic populations (Figure S2C). In all our differentiation experiments, unless otherwise stated, we did not use SB or CHIR for suppression of primitive haematopoiesis.

### 3.3 Transcriptome analysis of emerging haematopoietic populations

**FIGURE 4:**
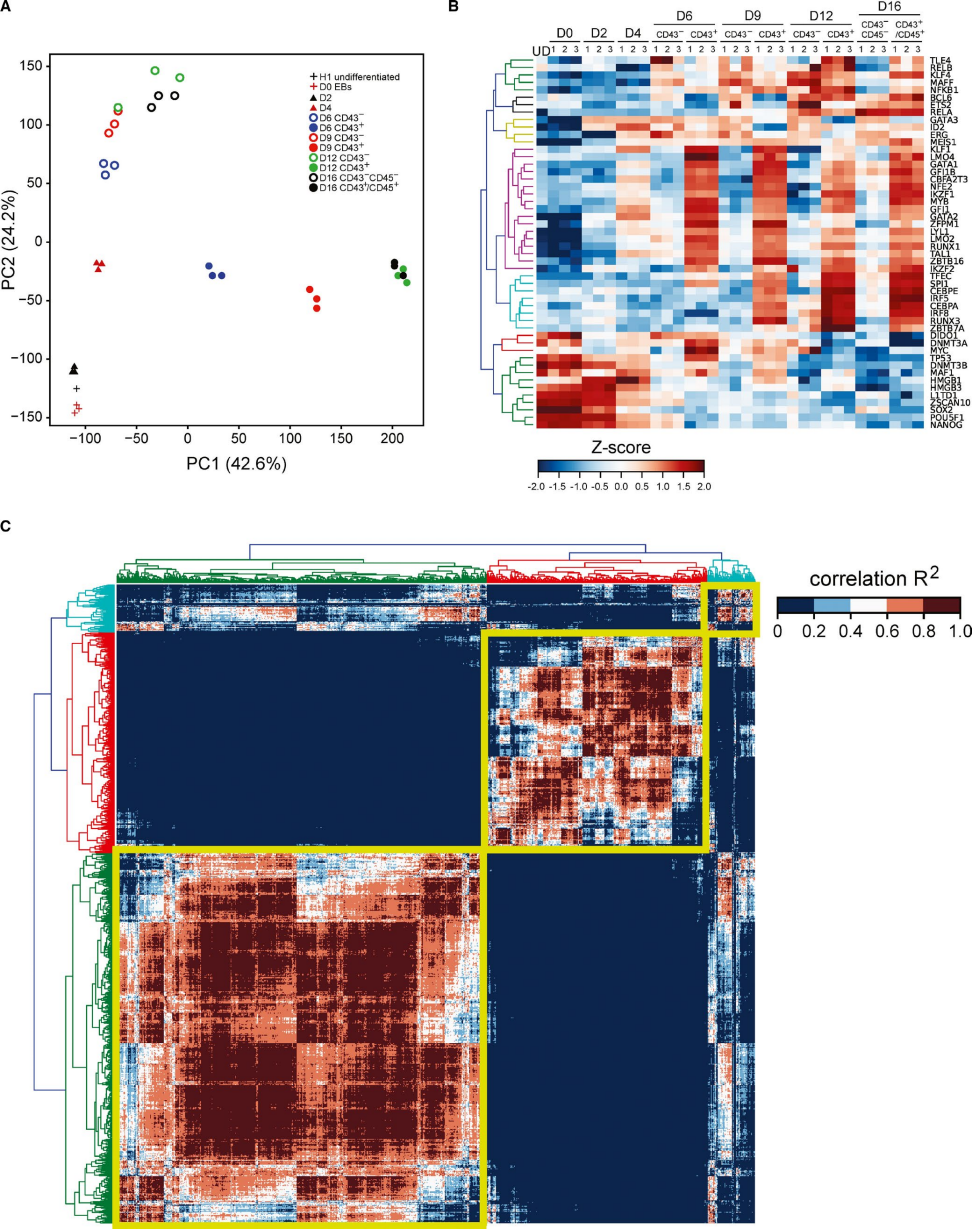
Global transcriptome analysis of the cytokine‐free differentiation system. (A) PCA of pooled RNA‐seq data reveals that CD43^+^ primitive blood specification starts from day 4 and continues until day 12 of H1 hESC differentiation. Three biological replicates were used for each cell population. (B) The induction of human haematopoiesis is accompanied by upregulation of key haematopoietic transcription factors whereas pluripotent genes are quickly suppressed. Gene expression levels in the heatmap here and elsewhere are normalized by a Z‐score transformation across the RNA‐seq experiments, with three independent biological replicates for each cell population. UD = undifferentiated H1 cells. (C). Global R‐squared multiple correlation analysis identifies three major superclusters of differentially expressed endothelial/mesenchymal, haematopoietic and cell migration genes

To evaluate the dynamics of gene expression during hPSC‐derived haematopoietic development, we performed the time‐course transcriptome analysis of CD43^+^ versus CD43^−^ cells. Principal component analysis (PCA) of differentially expressed genes (DEGs) demonstrated that CD43^+^ haematopoietic cells undergo gradually increasing and eventually strong segregation from the non‐haematopoietic lineages at the transcriptome level (Figure 4A). In line with this observation, all human haemoglobin genes were preferentially expressed in CD43^+^ haematopoietic cells (Figure S3A). The PCA revealed the progressive specification of CD43^+^ cells between day 4 and day 12 and showed that day 4 is the stage of segregation of the blood lineage. After day 12, no significant changes in the gene expression profile of haematopoietic cells were observed, which is in accord with the notion that the primitive haematopoietic development is time‐limited. A number of key haematopoietic transcription factors, such as LYL1, LMO2, RUNX1, TAL1, MYB1, GATA1, GATA2, GFI1, GFI1B, SPI1 were strongly and specifically upregulated in CD43^+^ cells. Some of these factors were upregulated already on day 4, while key pluripotency genes were strongly suppressed beyond that stage (Figure 4B). One hundred and forty‐eight genes out of the top 200 DEGs in the haematopoietic dimension (PC1+) were associated with active inflammatory Reactome pathways (Figure S3B). GO terms analysis of PC1+ DEGs also revealed strong domination of inflammatory trends (Figure S3C).

**FIGURE 5:**
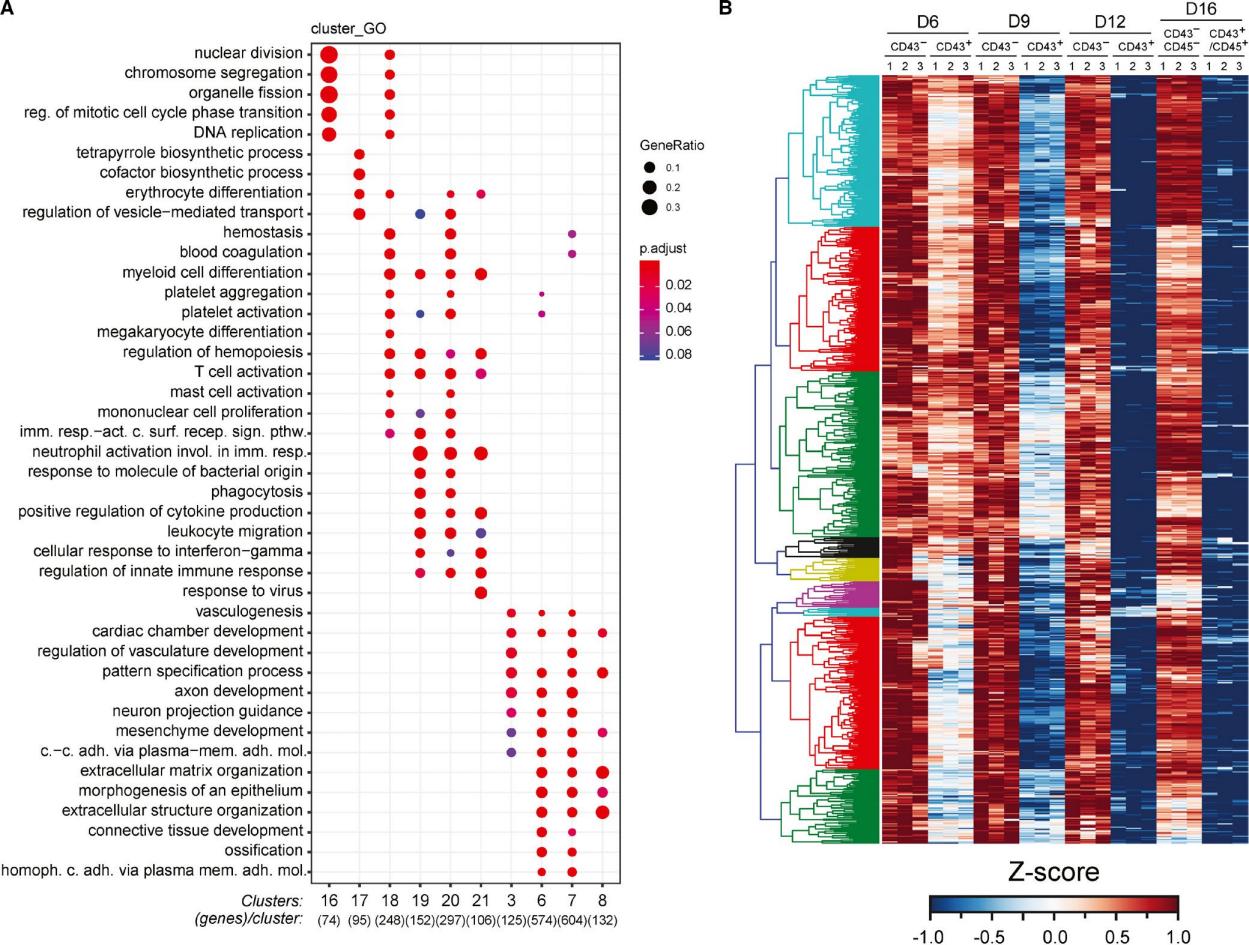
Transcriptome analysis of cell populations emerging during the course of the cytokine‐free differentiation. (A) Gene ontology diagram of the largest gene clusters that demonstrated the highest enrichment of the GO terms. (B) Heatmap diagram showing the dynamics of gene expression in the largest Cluster 7, which includes mostly endothelial and mesenchymal DEGs. The diagram shows the gene expression profile in CD43^+^ versus CD43^−^ cell populations throughout the key stages of the differentiation

For genome‐wide transcriptome analysis, we used the hierarchical clustering of the R‐squared multiple correlation matrix to identify three superclusters of differentially expressed genes (Figure 4C). The largest supercluster was comprised of mesenchymal and endothelial genes; haematopoietic genes formed the middle supercluster, and the smallest contained genes whose expression correlated with both large superclusters. The most enriched GO terms in the smallest supercluster were preferentially associated with cell migration (Figure S3D). The superclusters consisted of 40 clusters (clusters 0–39) of differentially expressed genes with a distinctive pattern of expression across eight blood and non‐blood cell populations. Clusters 3, 6–8 (C3, C6‐8) within the largest supercluster consisted of mesenchymal and endothelial genes preferentially upregulated in the CD43^−^ cells throughout the differentiation (Figure 5A and Figure S4). Consistent with the propensity of extraembryonic mesoderm for the production of extracellular matrix proteins,^4^ many genes of these clusters were involved in extracellular matrix generation and turnover (Figure 5A). The genes of C3 and the largest C7 were briefly upregulated on day 6 in CD43^+^ blood cells (Figure 5B and Figure S4). These clusters generated highly enriched GO terms associated with axonal and endothelial development highlighting common mechanisms and signals for growth, differentiation and guidance between neural and endothelial lineages.^27^ Thus, the early blood cells transiently express endothelial and angiogenic genes, which supports the notion that hPSC‐haematopoiesis, similar to the in vivo haematopoietic development, originates from the haemogenic endothelium (HE).

Practically all genes of major mesenchymal/endothelial clusters were strongly downregulated in CD43^+^ blood cells at advanced stages of differentiation (Figure 5B and Figure S4). In addition to the aforementioned upregulation of key haematopoietic transcription factors in the CD43^+^ fraction, this observation strongly suggests that the hPSC‐derived blood cells undergo faithful and determined haematopoietic specification that is unlikely to produce partially differentiated blood cells.

The largest clusters 16–21 within the haematopoietic supercluster incorporate genes upregulated in CD43^+^ blood cells (Figure S4). Seventy‐four genes of C16 were associated with the control of cell cycling and proliferation. Expression of the proliferative genes in blood cells reached a maximum on day 9 and was still upregulated later while CD43^−^ cells essentially turned off such genes on day 12. These data indicate that our differentiation system strongly and selectively promotes the proliferation of haematopoietic cells.

Enriched GO terms of C17 were related to the haem biosynthesis and metabolism in erythroid cells, erythrocyte differentiation and erythrocyte homeostasis (Figure 5B). The C17 genes were strongly expressed in day 6 and day 9 CD43^+^ cell populations reflecting the induction of erythroid specification at the early stages of the differentiation. C18 genes were upregulated at day 9 and the GO enrichment analysis indicated the preferential association of these genes with megakaryocyte and erythroid development and function. GO terms of cell cycling were also strongly represented in the cluster suggesting that C18 represents the transcriptome of the proliferative erythro‐megakaryocyte precursors. In sum, these data indicate that erythroid specification is initiated before the emergence of erythro‐megakaryocyte precursors.

The most enriched GO terms at the highest gene ratio in C19‐C21 referred to neutrophil activation involved in immune response and other aspects of inflammation such as the establishment of cellular response to interferons, positive regulation of cytokine production and phagocytosis (Figure 5A). C19‐C21 genes are most abundantly expressed in day 12 and day 16 CD43^+^ cells. These clusters include the top 183 DEGs strongly represented in GO terms associated with neutrophil activation (Figure S3C), which demonstrates the major inflammatory trend of blood cell specification during the cytokine‐free differentiation.

### 3.4 Broad developmental potential of early blood cells

**FIGURE 6:**
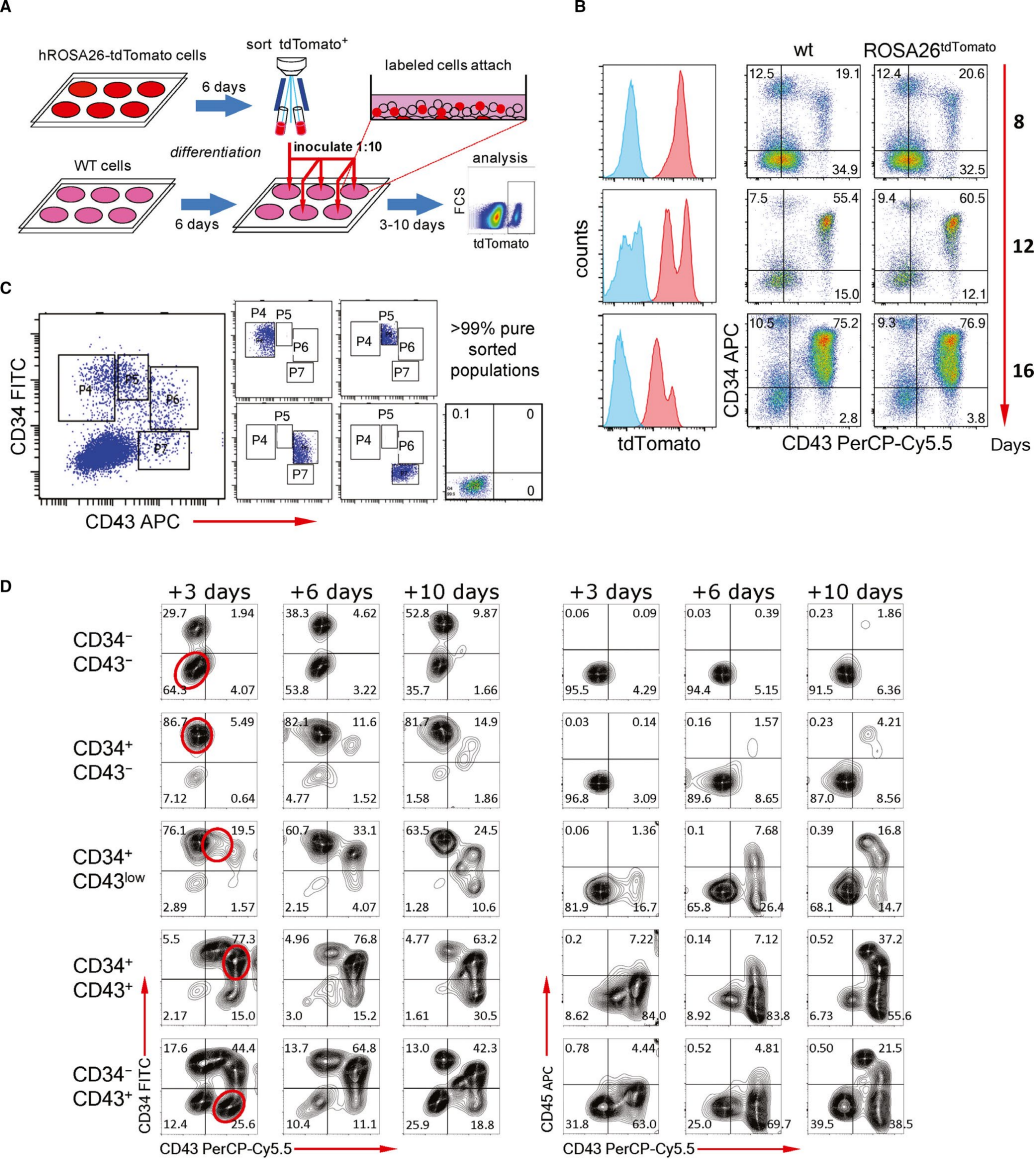
Strong transdifferentiation potential of the early human blood cells. (A) The outline of the in vitro cell tracing experiment. The sorted cells were returned into the same cellular context for realistic tracing of their further development. (B) The ROSA26^wt/tdTomato^ IPS12 cell line selected for the tracing experiments demonstrated ubiquitous expression of the reporter and normal haematopoietic development. Here and elsewhere, numbers in flow cytometry plots and histograms represent the percentages of cells within the respective quadrants or gated populations. (C) Scheme of CD34/CD43 cell sorting and the post‐sort analysis of the cell populations used for the tracing. (D) Representative (*n* = 3) developmental kinetics of the sorted populations at indicated time points of the chimeric culture. All flow cytometry panels show the phenotype of tdTomato^+^‐gated cells. Red open ellipses in the most left row of panels demonstrate the profile of the starting cell population

To delineate the developmental potential of emerging cell populations within the context of our differentiation system, we performed cell fate analysis in situ. To this end, hPSC‐derived haematopoietic cell populations labelled by a fluorescent marker were sorted and introduced into unlabelled synchronized differentiating cultures (Figure 6A). The chimeric cultures were continued for several days to trace the progeny of the labelled cells. For constitutive and ubiquitous labelling of hPSCs, we introduced the tdTomato reporter gene into the ROSA26 locus by TALEN‐mediated homologous recombination (Figure S5A). Correctly targeted clones were selected for the absence of off‐target events by Southern hybridization. The marker expression patterns of differentiated targeted clones and unmodified hPSCs were identical suggesting the absence of unspecific genetic alterations (Figure 6B and Figure S5B).

For the tracing, we sorted the early haematoendothelial cell populations characterized by differential expression of CD34 and CD43 (Figure 6C). The expression pattern of these key markers of early human haematopoiesis is remarkably similar in our system and other protocols.^22^, ^26^ Day 6 tdTomato^+^ individual populations were inoculated into same‐stage differentiation cultures of coisogenic unlabelled hPSCs and cultured for 3, 6 and 10 days before the phenotypical analysis. The developmental potential of the CD43^+^ early haematopoietic cells turned out to be dramatically higher compared to CD34^high^CD43^−^ endothelial/HE and CD34^−^CD43^−^ unspecified mesodermal cells (Figure 6D). The level of CD34 expression in CD43^+^ cells is inversely proportional to their developmental potential. The contribution of the endothelial/HE and mesodermal populations into haematopoiesis was negligible. CD34^high^CD43^−^ cell population has been reported to contain the definitive haematopoietic progenitors revealed by their T cell potential.^22^ The limited developmental potential of these cells demonstrates that definitive haematopoiesis is suppressed in our system, which preserves the definitive precursors from the premature specification. Indeed, CD34^high^ cells at the late stages of differentiation had a strong TCRαβ^+^CD3^+^ T cell potential (see below). The tracing data confirmed that the segregation of primitive blood is largely completed soon after its initiation between day 4 and day 6. In contrast, the generation of endothelial/HE/definitive precursors from CD34^−^CD43^−^ mesoderm continues several days longer. This is consistent with the dynamics of primitive versus definitive haematopoiesis in the mammalian conceptus.

To verify the ability of early human haematopoietic cells to transdifferentiate into endothelial and mesenchymal cells, we sorted day 12 CD43^+^ cells and seeded them on mCollagen IV in our defined VEGF‐containing differentiation medium supplemented by FGF2. In a few days of the secondary culture, the sorted cells started to adhere and proliferate, and after 2–3 weeks, three major cell fractions were detected: CD31^+^CD43^−^CD146^+^ endothelial cells, CD31^−^CD43^−^CD146^low/+^ mesenchymal and non‐adherent CD43^+^ haematopoietic cells (Figure S6). We compared the developmental potentials of CD43^+^ haematopoietic, CD31^+^CD146^+^ endothelial and CD31^−^CD146^+^ mesenchymal cells at day 12 of primary differentiation and found that only CD43^+^ haematopoietic cells gave rise to all three lineages after the secondary culture (Figure S6). In contrast, sorted mesenchymal CD31^−^CD43^−^CD146^+^ and endothelial CD31^+^CD43^−^CD146^+^ cells did not change their phenotype after prolonged secondary culture, eventually forming a monolayer of mesenchymal and endothelial cells, respectively. These findings are consistent with our observations made in the cell fate analysis. It is noteworthy that the progeny of day 12 CD43^+^ cells completely lost the endothelial and mesenchymal potential after 2 weeks culture in the serum‐free methylcellulose assay medium. Exogenous haematopoietic cytokines, therefore, eliminate the mesoangiogenic potential of the early blood. In sum, the removal of the cytokines revealed the full developmental potential of the early human haematopoietic cells.

### 3.5 Lineage specification in the cytokine‐free differentiation system

A recapitulative model of early human haematopoietic development is expected to generate specific human blood cell lineages that are detectable in the human conceptus. In the cytokine‐free system, CD235a^high^CD41^low/−^ erythroid cells emerged but failed to accumulate. Erythropoietin, EPO, is critically required for primitive human erythroblast development,^28^ and we tested whether the addition of this cytokine can support erythropoiesis in our cytokine‐free differentiation. Supplementing the primary differentiation with EPO led to a strong, concentration‐dependent expansion of CD235a^high^CD41^low/−^ primitive erythroblasts (Figure S7A) suggesting that the primitive human erythropoiesis can be effectively supported by a combination of VEGF and EPO. Furthermore, the rapid expansion of erythroid cells attests to the emergence of erythroid progenitors in the primary differentiation of hPSCs rather than during the clonogenic assay.

In contrast to the erythroid lineage, primitive megakaryocytes gradually accumulate in cytokine‐free conditions and undergo partial phenotypic maturation (Figure S7B). Myeloid cell lineages also sustain gradual maturation in the absence of exogenous cytokines. A large population of terminally differentiated CD11b^+^CD14^−/low^CD66b^+^ granulocytes, mostly neutrophils, arise by day 20, while CD11b^+^CD14^+^CD66b^−/low^ monocyte‐macrophages comprise about 1/3 of all CD45^+^ haematopoietic cells at this stage (Figure S7C). The strong bias towards myeloid lineage maturation is likely to be the result of the endogenous synthesis of the myeloid cytokines (Figure 3E). Notably, a number of neutrophil‐related genes were upregulated in CD43^+^ cells already on day 6 of differentiation (Figure 4A and Figure S3C).

**FIGURE 7:**
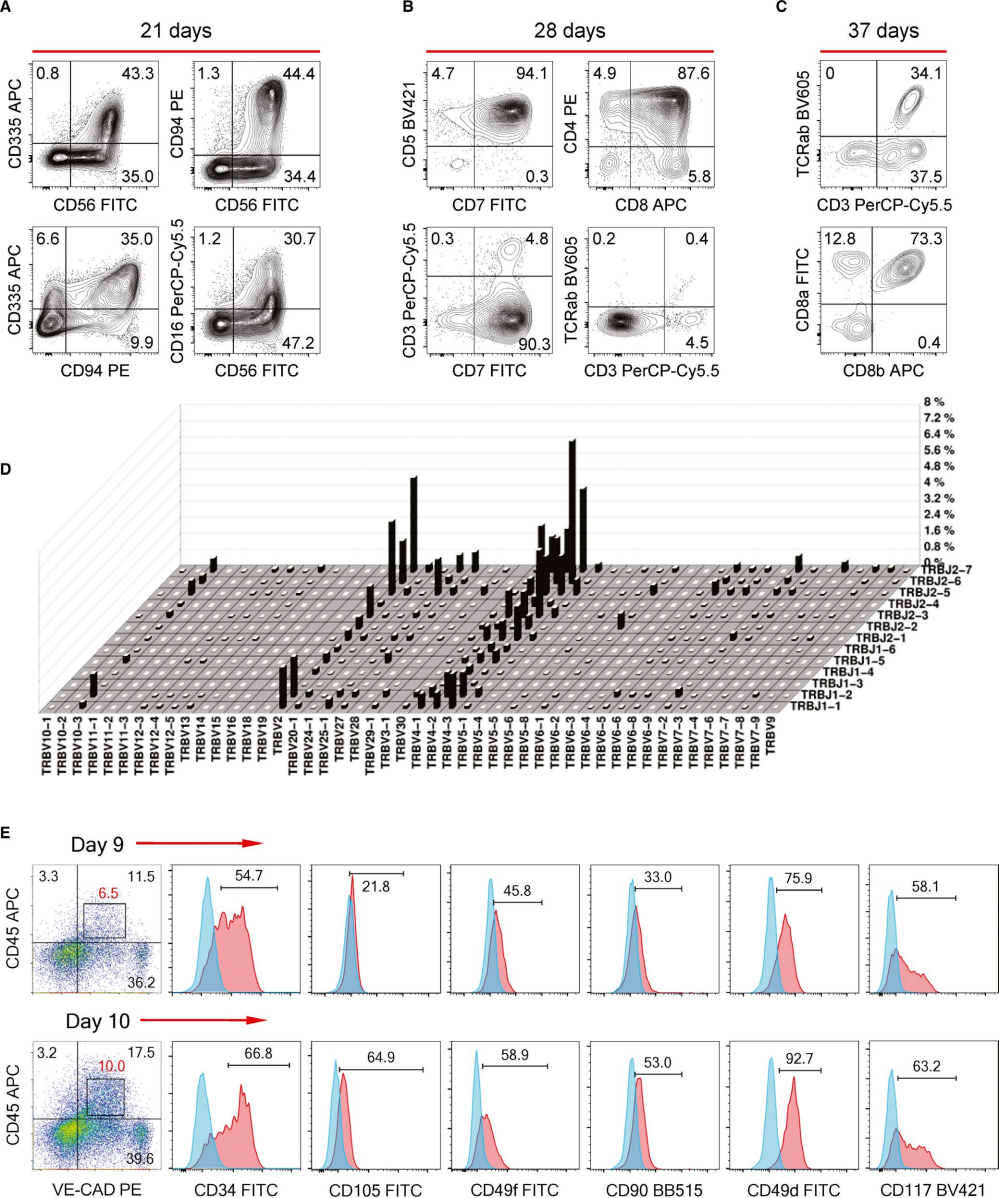
Development of the H1 hESC–derived definitive precursors into lymphoid cells and the early HSC‐like cells. The representative flow cytometry data of multiple lymphoid development experiments are shown. (A) The phenotype of NK cells developed from the primary day 6 CD34^+^CD43^–^ cells after 3 weeks of co‐culture with the OP9‐DL4 stroma. (B) Flow cytometry analysis of T cells developed from SB‐treated day 12 CD34^+^CD45^−^ primary cells after 4 weeks of the co‐culture. (C) After 37 days of the co‐culture, the hPSC‐T cells express significant levels of TCRαβ, CD3 and CD8b on their surface. (D) TCR‐seq diagram showing the spectra and the extent of V‐J rearrangements in the TCRβ locus after 5 weeks of the OP9‐DL4 co‐culture. (E) Flow cytometry analysis of Day 9 and Day 10 DP cells. Blue histograms represent the isotype control stainings

To test whether our protocol creates conditions for the generation of the lymphoid potential, we co‐cultured populations of the differentiated cells with OP9‐DL4 stroma in the presence of lymphopoietic cytokines. We found that day 6 CD34^+^CD43^−^ cells were able to generate lymphoid cells after a 5 week co‐culture while CD34^low^CD43^+^ cells completely lacked the lymphoid potential (Figure S7D). Day 6 CD34^+^CD43^−^ cells predominantly generated NK cells in the lymphoid differentiation conditions (Figure 7A). These NK cells were extremely cytotoxic killing the entire OP‐DL4 stroma within 11–14 days of the co‐culture. Many of these NK cells demonstrated high levels of CD16 and CD335 (NKp46) expression, which correlated with the observed intense cytotoxicity.^29^, ^30^ When we treated the primary differentiation with SB to suppress primitive haematopoiesis, both CD34^+^CD45^−^ and CD34^+^CD45^+^ cell populations at day 12 to day 16 demonstrated a robust T cell potential (Figure 7B), which suggests the efficient maintenance of CD34^+^ T cell precursors in the cytokine‐free conditions. A large population of CD4^+^CD8^+^ T cells emerged within 4 weeks while longer co‐culture led to an increasingly strong expression of key functional markers CD3, CD8b and TCRαβ on the surface of these cells (Figure 7C). TCR‐seq analysis of the non‐adherent cell fraction of the 5 week OP9‐DL4 co‐culture showed intense V‐J rearrangements in the TCRβ locus suggesting the functionality of the hPSC‐T cells (Figure 7D).

Another recapitulative feature of our differentiation system is the emergence of CD45^+^VE‐CADHERIN^+^ double‐positive (DP) cell population that is phenotypically similar to early human embryonic HSCs. Such DP HSCs were found in the aorta‐gonad‐mesonephros region and the embryonic liver.^31^, ^32^ We detected the emergence of the DP population starting on day 9 of the SB‐free H1 and IPS12 differentiation. Similar to the embryonic DP HSCs, the hPSC‐DP cells expressed low levels of VE‐CADHERIN and CD45 while endothelial and myeloid cells expressed these markers, respectively, at the levels of about 10 times higher (Figure S7E). The hESC‐DP cell population co‐segregated with a vast majority of the primitive clonogenic progenitors, and practically all CFU‐mix^P^ progenitors had the DP phenotype (Figure S7D). In addition, cells within the DP population expressed a number of human HSC markers, such as CD34, CD49d, CD49f, CD90, CD105 and CD117 (Figure 7E). Similar to their embryonic counterparts, the hPSC‐DP cell population was transitory; the numbers of these cells decline significantly by day 12 of H1 hESC differentiation. Strong clonogenicity and the HSC‐like phenotype of the DP cells suggest that the population may contain haematopoietic progenitors with the engraftment potential.

Collectively, our data indicate that VEGF‐driven, cytokine‐free haematopoietic differentiation of hPSC efficiently produces cells of all myeloid lineages as well as erythroid, T, and NK cell precursors together with progenitors that are phenotypically similar to human embryonic HSCs.

## 4 DISCUSSION

In this work, we found that sustainable haematopoietic development of hPSCs can be achieved in defined culture conditions and in the absence of exogenous haematopoietic cytokines. Our data demonstrate that VEGF and endogenous stimuli are sufficient to drive robust haematopoietic development, generation of clonogenic progenitors and definitive haematopoietic precursors. Moreover, the addition of cytokines to hPSC differentiation turned out to detrimental for the progenitor generation. The minimalistic approach employed in our system ensured reproducible hPSC differentiation, which is important for functional genetic studies. The novel differentiation protocol creates a system for studying the regulation of the early human haematopoietic development. Important features of our system are simplicity, efficiency, low cost, suitability for biomedical applications and closer recapitulation of the early haematopoietic development.

Several recapitulative features distinguish our system from the existing protocols. Similar to the morphological features of the yolk sac haematopoiesis,^33^ the hPSC‐derived endothelial/angioblastic cords precede the emergence of blood island structures, the IBIs, during induction of haematopoiesis. Regularly spaced, occasionally fused IBIs locate around the site of EB attachment and vividly mimic the yolk sac blood islands in mammalian conceptuses. Similar to the in vivo blood islands, the IBIs were transient structures quickly losing their cellular content through EHT. Another recapitulative feature of the protocol is the emergence of the DP progenitor population that is phenotypically similar to early embryonic human HSCs. We also detected at later stages of differentiation a strong upregulation of genes that were associated with the activation of inflammation. The involvement of the inflammatory signalling in the midgestation haematopoietic development has been previously demonstrated.^34^

The observed transdifferentiation potential of hPSC‐blood cells has to be assessed against the possibility of ‘incomplete differentiation’ of hPSC‐derived cells.^35^ Our transcriptome analysis revealed that the hPSC‐derived haematopoietic cells strongly suppressed endothelial and mesenchymal genes at the stages at which they demonstrate their transdifferentiation potential. This finding indicates the determined haematopoietic specification that is unlikely to produce incompletely differentiated blood cells. Therefore, the in vitro haematopoietic cells demonstrated the mesoangioblastic potential, which was reported for the early haematopoietic cells in the embryo.^36^, ^37^ The yolk sac–derived haematopoietic cells demonstrated their mesoangioblastic potential after their migration into the embryo proper.^38^

## 5 CONCLUSION

In conclusion, we developed and characterized an advanced recapitulative protocol for haematopoietic differentiation of hPSCs in defined cell culture conditions and the absence of exogenous haematopoietic cytokines. We propose the cytokine‐free differentiation of hPSCs as a system of choice for studying early human haematopoietic development.

## CONFLICT OF INTEREST

The authors declared no potential conflicts of interest with respect to the research, authorship, and/or publication of this article.

## AUTHOR CONTRIBUTION

**Elena S Philonenko:** Data curation (equal); Formal analysis (equal); Investigation (lead); Methodology (lead); Validation (equal); Visualization (equal); Writing‐review & editing (equal). **Cuihua Wang:** Data curation (supporting); Formal analysis (equal); Investigation (equal); Methodology (equal); Resources (supporting); Software (lead); Validation (supporting); Visualization (equal). **Ying Tan:** Conceptualization (supporting); Data curation (supporting); Formal analysis (equal); Investigation (lead); Methodology (lead); Visualization (equal); Writing‐review & editing (equal). **Baoyun Zhang:** Investigation (equal); Methodology (equal); Resources (equal); Validation (supporting); Writing‐review & editing (supporting). **Zahir Shah:** Formal analysis (supporting); Investigation (equal); Methodology (equal); Software (supporting); Validation (supporting). **Jianguang Zhang:** Investigation (equal); Methodology (equal); Visualization (supporting). **Hanif Ullah:** Investigation (equal); Methodology (equal). **Sergei L. Kiselev:** Resources (equal); Supervision (supporting); Writing‐review & editing (supporting). **Maria Lagarkova:** Resources (equal); Supervision (supporting); Writing‐review & editing (supporting). **Dandan Li:** Funding acquisition (supporting); Resources (equal). **Yong Dai:** Formal analysis (supporting); Funding acquisition (supporting); Resources (supporting); Supervision (supporting); Writing‐review & editing (supporting). **Igor M. Samokhvalov:** Conceptualization (lead); Data curation (lead); Formal analysis (lead); Funding acquisition (lead); Investigation (equal); Methodology (equal); Project administration (lead); Resources (equal); Supervision (lead); Validation (lead); Visualization (equal); Writing‐original draft (lead); Writing‐review & editing (lead).

## SUPPLEMENTAL MATERIALS AND METHODS

Supplemental materials and methods for this article are available online https://wiley.eproofing.in/OPS/20210727062001182/ProofLink2/supinfo/jcmm16826‐sup‐0001‐Supinfo.pdf.

## Supporting information

Supplementary Material

## Data Availability

The RNA‐seq data have been deposited with Gene Expression Omnibus under the accession number GSE159672. All other relevant data are available from the corresponding author upon request.
