# Supplementary material for: Recapitulative haematopoietic development of human pluripotent stem cells in the absence of exogenous haematopoietic cytokines
Source: J Cell Mol Med. 2021 Aug 2;25(18):8701–14. doi: 10.1111/jcmm.16826 (PMC8435420; doi:10.1111/jcmm.16826)
Supplement: Supplementary file 1 — Supplementary Material [file JCMM-25-8701-s001.pdf]

Supplemental materials and methods

Culturing hPSCs

All hESC (H1/WA01, HN14, MEL1) and hiPSC (iPS12, iPSS1, and iPSS9) lines were maintained in the undifferentiated state on Matrigel-coated plates (Corning Matrigel, Cat. No. 354230) in mTeSR1 medium (STEMCELL Technologies, Vancouver, Canada). The cell lines were regularly tested for mycoplasma contamination. Normally, no antibiotics were added to the hPSC cultures to facilitate timely exposure of any mycoplasma contamination. To improve cell survival, the culture medium was supplemented with 1 μM Thiazovivin (GIBH CAS, China) during the first 24 hours of post-thaw recovery. A single-cell suspension of hPSCs was obtained by dissociation of 70-80% confluent hPSC cultures with TrypLE™ Express (Thermo Fisher Scientific, Waltham, MA) for a minimum time period at 37^0C. Before transfection and hematopoietic differentiation, hPSCs were subjected to at least three short passages (for 2-3 days) at a seeding density of $4\text{-}6\times10^6$ cells per one well of a standard 6-well plate. The cells were maintained at 37^0C in humidified normoxic conditions in a 5% CO_2 atmosphere.

Hematopoietic differentiation of hPSCs

To initiate hematopoietic differentiation, exponentially growing cultures of hPSCs were detached into a single-cell suspension by TrypLE™ Express. For EB formation, usually, 1×10^6 single cells were spun at 100×g for 4 min into AggreWell™400 (STEMCELL Technologies) in mTESR1 medium containing 1 μM Thiazovivin and incubated for 20-24 hours at 37^0C and 5% CO_2. The newly formed EBs were carefully flushed out of AggreWells and filtered through BD Falcon™ 40 μm Cell Strainers to remove unincorporated single cells. The purified EBs then were induced to attach to Corning® mCollagen IV (Corning Life Sciences, Bedford, MA) –

coated surfaces under normal gravity in mTeSR1 medium supplemented with 2-4 ng/mL hBMP4 (PeproTech, Rocky Hill, NJ), 50 ng/mL $hVEGF_{165}$ (PeproTech), and 10 μM Thiazovivin. For coating, the concentration of Collagen IV was adjusted to 0.5 $\mu g/cm^2$ by dilution in 50 mM HCl. The optimal density of seeding on the Collagen IV layer was 150-200 EB clumps per one well of the 6-well plate. The day of the start of EB attachment was considered as Day 0 of differentiation.

After 48 hours of the EB attachment, the medium was replaced with pre-warmed 2 mL/well of the Hematopoietic Medium [StemLine II HSC expansion medium (Sigma-Aldrich, St. Louis, MO), 1×GlutaMax (Thermo Fisher Scientific), 1×2-Mercaptoethanol (2-ME, Sigma-Aldrich), 1×NEAA (non-essential amino acids, Thermo Fisher Scientific), and 50 ng/mL $hVEGF_{165}$ (Peprotech)]. Two days after the medium replacement, 2 mL of fresh Hematopoietic Medium was added to each well. Onward, half of the medium, 2 mL per well, was replaced with fresh Hematopoietic Medium every other day until analysis.

To modulate primitive versus definitive hematopoiesis, SB-431542 (TOCRIS, Bio-Techne, Minneapolis, MN) and CHIR99021 (STEMCELL Technologies), an agonist of the WNT signaling, were added to Day 2 - Day 4 hESC differentiating cultures at a final concentration of 2 to 6 μM, and 3 μM, respectively. Activin A (PeproTech) was added to a final concentration of 1 ng/mL on Day 0 - Day 2 of differentiation.

ROSA26 targeting

The best pair of TALENs was selected using the GFP reporter rescue assay.[1] In a typical transfection experiment, 1×10^6 cells were electroporated with 10 μg of the linearized targeting construct and 10 μg of each TALEN plasmid in mTeSR1 medium containing 1 mM Thiazovivin

in two consecutive pulses at 240 V and 500 μF. After electroporation, the cells were seeded on a Matrigel-coated 6-well plate in mTeSR1 + 1 μM Thiazovivin at a density of 2.5×10^5 cells/well. The medium was changed daily and drug selection was performed with 500 ng/mL of puromycin after 48-72 hours of post-electroporation recovery. To control the acquisition of the drug resistance, the same procedure was performed in the same conditions without the targeting construct. Typically, the drug selection continued for 3-4 days until all cells perished in a control dish and individual puromycin-resistant colonies appeared in the targeted sample. The colonies were grown until they reached the size of ~1000 cells/colony. Individual colonies were picked up under a microscope in an aseptic environment, dissociated briefly by TrypLE, and further cultured in individually-labeled Matrigel-coated wells of 24-well plates in mTeSR1 + 1 μM Thiazovivin. Genomic DNA of the targeted hESC clones was isolated by Proteinase K digestion followed by phenol-chloroform extraction.

To delete the floxed PGK-$Puro^R$ gene cassette, we transfected correctly targeted clones with Cre mRNA followed by the duplicate selection of $Puro^S$ clones. The selected clones were picked, expanded, and analyzed as described above. Cre mRNA was synthesized and tailed with polyA using HiScribe™ T7 ARCA mRNA kit (Cat. No. E2060S, New England BioLabs, Ipswich, MA) according to the manufacturer's instructions.

Southern blot hybridization

Southern blotting was performed according to a protocol supplied by the manufacturer of $Nylon^+$ membrane (BrightStar™-Plus, Invitrogen by Thermo Fisher Scientific, Waltham, MA) with some modifications. The DNA probe labeling and chemiluminescent signal development were carried out with the use of the North2South Biotin Random Prime Labeling Kit (Thermo Fisher

Scientific, Cat. No. 17075) according to the manufacturer's recommendations. Hybridization was performed in the Church-Gilbert buffer at 65^0C. The images were captured by ChemiScope 5300 (CLiNX Science Instruments Co., Shanghai, China).

Flow cytometry and cell sorting

For the cell flow cytometry and sorting, we used anti-human monoclonal antibodies from Becton Dickinson (BD Life Sciences, Franklin Lakes, New Jersey). All cell sorting procedures were performed either on BD FACSAria II or Beckman Coulter MoFlo Astrios machines. The flow cytometry analyses were done on BD C6 Accuri and BD LSRFortessa. The data were analyzed with FlowJo V10 (FlowJo LLC, BD) and BD C6 Accuri software.

For flow cytometry analysis, cells were harvested using the standard TrypLE procedure followed by the removal of the remaining cell/extracellular matrix clumps by the Cell Strainer. The cells were centrifuged at 300×g for 5 min at room temperature and the pellet was resuspended in the Hematopoietic Medium (StemLine II, 1×GlutaMax, 1×2-ME, 1×NEAA). The resulting cell suspension was incubated in CO_2-incubator with the loosened cap for at least 1 hour to restore TrypLE-sensitive antigens. Next, the cells were centrifuged as described above and resuspended in the cold FACS Buffer (1×D-PBS-Ca-Mg, 5% FCS, 20 mM HEPES pH 7.2-7.5) containing 5% normal human serum at a density of 1×10^6 cells per 100 μL and incubated on ice for at least 10 min. After the serum blocking, the cells were incubated with specific antibodies for 20 min on ice in the dark. The unbound antibodies were washed twice with 1 mL of cold FACS Buffer and spun down at 300×g for 5 min at 4^0C. For gating out dead or apoptotic cells the washed cell suspensions were incubated with 7-AAD (BioLegend, San Diego California) for analysis by BD C6 Accuri or DAPI for the BD LSRFortessa cytometry.

For sorting, cells were harvested, incubated with antibodies, and washed as described above. For cell sorting staining we routinely used 5×BD test amount of antibodies per 1×10^7 cells and increased the incubation time to 40 min. After the final wash, the cells were resuspended in 500 µL – 1 mL of the FACS Sorting Buffer (1×Han's Balanced Salt Solution, 2% BSA (Sigma-Aldrich), 25 mM HEPES pH7.2-7.5, 1mM EDTA). The cells were filtered through the 40 µm Cell Strainer, sorted into the Basal Hematopoietic Medium, and washed twice by DMEM/F12 medium (Thermo Fisher Scientific) before downstream applications.

Immunocytofluorescence

For immunocytochemical studies, cells were fixed in 4% paraformaldehyde for 20 min at room temperature. Fixed cells were incubated for 1 hour in the Blocking Buffer (1×D-PBS, 0.2% Triton X 100, 0.1% Tween-20, 2% goat or donkey serum, and 2% FBS). Primary antibodies were diluted in 1×D-PBS, 0.1% Tween-20, and applied for 1–3 hours at room temperature or overnight at 4^0C. Slides were washed 3×3 min with 1×D-PBS – 0.1% Tween-20 solution, and incubated with Alexa Fluor 546- or Alexa Fluor 488-conjugated either goat anti-mouse or goat anti-rabbit or donkey anti-goat (all of Invitrogen brand, Thermo Fisher Scientific) secondary antibodies used at a dilution of 1:1000. Nuclei were stained with DAPI (Sigma-Aldrich). Slides were mounted in the Vectashield® mounting medium (Vector Laboratories, Burlingame, CA) and examined under the confocal microscope LSM800 (Carl Zeiss AG, Oberkochen, Germany).

Hematopoietic progenitor assay

Hematopoietic progenitor assay was performed in the serum-free methylcellulose medium SF H4436 (STEMCELL Technologies) according to the manufacturer's recommendations in duplicates for at least two different cell densities of each input cell population ranging from

5×10^3 to 5×10^4 cells per 1.5 mL of the H4436 medium in a 35 mm Petri dish. The colonies were grown for 16-18 days at 37^0C in the 5% CO_2 atmosphere. The colonies were scored under high magnification (100×) and the hematopoietic progenitors were retrospectively classified into different types of Colony Forming Unit - Cells (CFU-Cs): BFU-E^P (Burst Forming Unit – Erythroid, primitive), CFU-E^P (Colony Forming Unit – Erythroid,pPrimitive), CFU-Myeloid (Colony Forming Unit – Macrophage, Granulocyte, and Granulocyte-Macrophage), CFU-Mix^P (Colony Forming Unit – Mixed lineage, primitive). May-Grünwald histochemical staining of individual colonies was performed to confirm the colony identity.

May-Grünwald staining

Sorted cells ($0.5\text{-}1.0\times10^5$) were washed and resuspended in 100 µL 1×D -PBS. Cells were spun onto polylysine-coated slides at 500 rpm for 5 min, air-dried, and stained with May-Grünwald stain (Sigma-Aldrich, MG500) according to the manufacturer's protocol. The slides were washed with dH_2O, air-dried, and mounted for examination by light microscopy.

Quantitation of cytokine synthesis

Hematopoietic cytokines in the cell culture supernatants were quantitated by Quantikine ELISA kits, R&D Systems™ (Fisher Scientific, Thermo Fisher Scientific), according to the manufacturer's instructions. The following R&D Systems™ kits were used: hIL-1β, Cat. No. DLB50; hIL-8, Cat. No. D8000C; hIL-10, Cat. No. D1000B; hIL-11, Cat. No. D1100; hIL-16, Cat. No. D1600; SCF, Cat. No. DCK00; hM-CSF, Cat. No. DMC00B.

Lymphoid cell development

For NK cell generation, Day 6 total primary differentiated cells were harvested with TrypLE and $CD34^{high}CD43^{-}$ cells were isolated using magnetic-activated cell sorting (MACS) with CD43 (first negative selection) and CD34 (second positive selection) MicroBeads, human, (Miltenyi Biotec, Auburn, CA) according to the manufacturer's protocol.

For T cell generation, Day 12 SB-treated cells of the primary hematopoietic differentiation were harvested, and $CD34^{+}CD45^{low/-}$ cells were isolated by MACS using CD34 and CD45 MicroBeads, human, (Miltenyi Biotec), while $CD34^{+}CD45^{+}$ cells were purified by FACS as described above.

To generate stromal cells expressing DL4, low-passage OP9 cells were transfected with a PiggyBac transposon vector bearing human *DLL4* cDNA under the EF-1α promoter and a $Puro^{R}$ gene cassette. Puromycin-resistant OP9-DL4 cells were selected and maintained in α-MEM supplemented with 20% FCS (Thermo Fisher Scientific). A total of $2–5×10^{5}$ cells of the MACS-enriched cell populations were added to the individual well of a 6-well plate containing OP9-DL4 cells and cultured in the OP9 differentiation medium (α-MEM, 20% FBS, and 50 μM 2-mercaptoethanol) supplemented with rhSCF (5 ng/mL), rhIL-7 (5 ng/mL), and rhFLT3-L (5 ng/mL) (PeproTech). Every 7 days, differentiating human cells were placed on the fresh OP-DL4 stroma. Human PSC-derived NK cells were usually collected after 3 weeks of the co-culture, while T cells were cultured for 5-6 weeks.

TCR repertoire sequencing

Total non-adherent and loosely adherent cells after 5 weeks of the T cell differentiation were collected, centrifuged, and cell pellets were frozen in the TRIzol Reagent (Thermo Fisher

Scientific). The sequencing and analysis of TCRβ CDR3 regions were performed by Novogene (Beijing, China).

Transcriptional profiling by RNA sequencing

H1 hESCs at various stages of the hematopoietic differentiation were used for RNA sequencing as follows: unsorted total live cells for undifferentiated hESCs and differentiated cells at Day 0, 2, 4; sorted $CD43^{+}$ and $CD43^{-}$ cell populations at Day 6, 9, 12; $CD43^{-}CD45^{-}$ and $CD43^{+}/CD45^{+}$ populations at Day 16 of differentiation. Three independent biological repeats were prepared for each cell population at each differentiation stage totaling 34 samples. Samples were sent for RNA sequencing at RiboBio (Guangzhou, China) and Annoroad Gene Technology (Beijing, China). As a quality control step, the RNA-seq data were analyzed for normal gene expression, using the previously described method.[2,3] In brief, sequenced reads were aligned to the genome using bowtie2[4] and RSEM[5], with the settings '—bowtie2—bowtie2-sensitivity-level very_sensitive— no-bam-output—estimate-rspd' using an index built against the Ensembl annotations (v81), and normalized using EDAseq[6] (v2.4.1) (which = 'full'). RNA-seq data is expressed in units of GC-normalized tag counts. Differential expression was called using DESeq2[7], q < 0,05, and fold change of >4 were used as the threshold. For GO enrichment, we performed a gene set over-representation test on cluster signature genes using R package clusterProfile with Reactome (https://reactome.org/) database. Another analysis was performed using glbase3.[8] The RNA-seq data have been deposited with Gene Expression Omnibus under the accession number GSE159672.

Supplementary References

1. Ma N, Liao B, Zhang H, et al. Transcription activator-like effector nuclease (TALEN)-mediated gene correction in integration-free β-thalassemia induced pluripotent stem cells. J Biol Chem 2013;288:34671-34679.
2. Hutchins AP, Takahashi Y, Miranda-Saavedra D. Genomic analysis of LPS-stimulated myeloid cells identifies a common pro-inflammatory response but divergent IL-10 anti-inflammatory responses. Sci Rep 2015;5:9100.
3. Hutchins AP, Yang Z, Li Y, et al. Models of global gene expression define major domains of cell type and tissue identity. Nucleic Acids Res 2017;45:2354-2367.
4. Langmead B, Salzberg SL. Fast gapped-read alignment with Bowtie 2. Nat Methods 2012;9:357-359.
5. Li B, Dewey CN. RSEM: accurate transcript quantification from RNA-Seq data with or without a reference genome. BMC Bioinformatics 2011;12:323.
6. Risso D, Schwartz K, Sherlock G, Dudoit S. GC-content normalization for RNA-Seq data. BMC Bioinformatics 2011;12:480.
7. Dobin A, Davis CA, Schlesinger F, et al. STAR: ultrafast universal RNA-seq aligner. Bioinformatics 2013;29:15-21.
8. Hutchins AP, Jauch R, Dyla M, Miranda-Saavedra D. glbase: a framework for combining, analyzing and displaying heterogeneous genomic and high-throughput sequencing data. Cell Regen 2014;3:1.

Supplemental Figure 1. Planar cytokine-free differentiation of hPSCs on extracellular matrix proteins.

(A) In the novel differentiation system, H1 hESC differentiation on Fibronectin and Tenascin C did not show a detectable advantage over mCollagen IV in terms of the development of the key hematopoietic populations.

(B) Flow cytometry reveals gradual segregation of the three major cell lineages emerging during the hPSC differentiation. On Day 16, cell staining with CD31/CD43/CD146 antibodies clearly shows the differential phenotypes of $CD31^{+}CD146^{+}$ endothelial cells, $CD31^{-}CD146^{+/low}$ mesenchymal cells, and $CD31^{low}CD43^{+}$ blood cells. Here and elsewhere, numbers in flow cytometry plots represent the percentages of cells within the respective quadrants.

(C) The kinetics of CD45 cell surface expression on H1 hESC-derived hematopoietic cells at the indicated time points.

(D) Addition of FGF2 at 5 and 20 ng/mL on Day 0 - 4 suppresses the hPSC-derived hematopoiesis. Representative data for H1 hESCs is shown.

Supplemental Figure 2. The efficiency of the hematopoietic differentiation among different lines of hPSCs is variable, while Activin signaling inhibition as well as induction of WNT signaling suppressed the hPSC-derived primitive hematopoiesis.

(A) Time course analysis of blood marker expression during the cytokine-free differentiation of the indicated hPSC lines.

(B) Efficiency of hematopoietic progenitor generation by the hPSC lines.

(C) SB-431542, an Activin/Nodal signaling inhibitor, and CHIR99021, an agonist of WNT signaling, strongly suppressed the emergence of primitive blood cells by differentiating cultures of H1 ESCs. CD43$^{-/low}$CD34^{+} cell population that included definitive hematopoietic precursors persisted in Day 12 SB-treated cultures.

Supplemental Figure 3. Expression of CD43 effectively separates non-hematopoietic lineages from hematopoietic cells that upregulate inflammatory genes during the course of the differentiation.

(A) Hemoglobin mRNA expression in CD43^{+} versus CD43^{-} cells at Day 6, 9, and 12 of differentiation and in undifferentiated (UD) H1 cells. Data are mean ± SEM, n = 3.

(B) Human Reactome pathways associated with top 200 DEGs of the two principal component dimensions in the PCA.

(C) Gene ontology terms associated with top 200 DEGs of the two principal component dimensions in the PCA.

(D) The minor supercluster of the global R-squared multiple correlation plot consists of mainly migratory genes.

Supplemental Figure 4. Expression dynamics of DEGs from the major gene clusters presented in Figure 5A.

The gene expression dynamics in CD43^{+} versus CD43^{-} cells are displayed as cluster heatmap diagrams. Each heatmap is annotated at the top, gene names are now shown due large numbers of genes in each subcluster. The heatmaps for six hematopoietic clusters (Clusters 16-21) are

shown at the top. Gene expression levels in the heatmaps are normalized by Z-score transformation across the RNA-seq experiments, with three independent biological repeats for each cell population. UD = undifferentiated H1 hESCs.

Supplemental Figure 5. Constitutive and ubiquitous reporter cell line for cell tracing studies *in vitro.*

(A) Scheme of TALEN-mediated *ROSA26* knock-in gene targeting in IPS12 cells and Southern hybridization screening of correctly targeted clones with external and internal DNA probes. Exons are open rectangles, the *ROSA26* promoter is shown as a black oval, *loxP* sites are designated by black triangles. *En2-SA* is a splice acceptor fragment of the mouse *Engrailed-2* gene that was inserted into the targeting construct to facilitate splicing of the knock-in transgene. Positions of two DNA probes for the Southern blot hybridization and the size of the indicative DNA fragments are shown along WT and the targeted allele. The deletion of the PGK-Puro^{R} gene cassette in the *ROSA26* KI allele by Cre recombinase produces the working configuration of the targeted allele.

(B) The differentiation quality of the $ROSA26^{WT/tdTomato}$ cell line selected for *in vitro* cell tracing was confirmed by flow cytometry with the antibodies to the indicated hematopoietic markers.

Supplemental Figure 6. Comparative analysis of the development potential of blood and non-blood cell lineages generated by the cytokine-free differentiation of H1 hESCs.

On Day12 of the differentiation, $CD43^{+}CD146^{low/-}$ blood cells, $CD31^{+}CD146^{+}$ endothelial cells, and $CD31^{-}CD146^{+}$ mesenchymal cells were sorted and cultured separately for 2-3 weeks in the same conditions except that FGF2 was included in the medium. In contrast to the mesenchymal and endothelial cells, the blood cells generated both the adherent and non-adherent cell fractions. The blood-derived adherent fraction consisted of mesenchyme and endothelial cells suggesting the transdifferentiation process. The scheme delineates a compilation of more than 10 experiments. Cell sorting and flow cytometry plots show the representative data. Scale bars – 40 μm (adherent cells); 100 μm (tube formation panels).

Supplemental Figure 7. Maturation of blood cell lineages and characterization of hPSC-derived DP cells and lymphoid precursors.

Representative flow cytometry data are shown.

(A) Flow cytometry analysis of EPO inducing the erythroid maturation in the primary differentiation culture of H1 hESCs. The analysis was performed on Day 10 of the differentiation. The cytospin staining panel shows the phenotype of $CD235a^{+}CD41a^{low}$ erythroid cells (Day 10, 6 U/mL of EPO) which were sorted as designated by open red ovals on the cytometry plots. Scale bar – 25 μm.

(B) The time-course of the spontaneous maturation of the H1-derived megakaryocyte lineage with corresponding May-Grünwald staining of the Day16 $CD42b^{+}CD61^{+}$ cell population shown by the red open rectangle. Scale bar –25 μm.

(C) Spontaneous maturation of monocyte/macrophages and granulocytes during the primary differentiation of H1 cells. Two panels with corresponding cytospin staining of sorted cell

populations are shown below. The sorting gates are designated by open red and blue rectangles in the CD14/CD66b flow cytometry plot. Scale bars – 25 μm.

(D) hPSC-derived $CD34^{low}CD43^{+}$ primitive blood cells do not have the lymphoid potential.

(E) The DP cell population contains the majority of clonogenic hematopoietic progenitors. Day 12 IPS12 cells were sorted as shown in the left panel and seeded in the methylcellulose assay medium to assess the density of clonogenic progenitors, the right panel. The data are mean ± SD, n = 4.

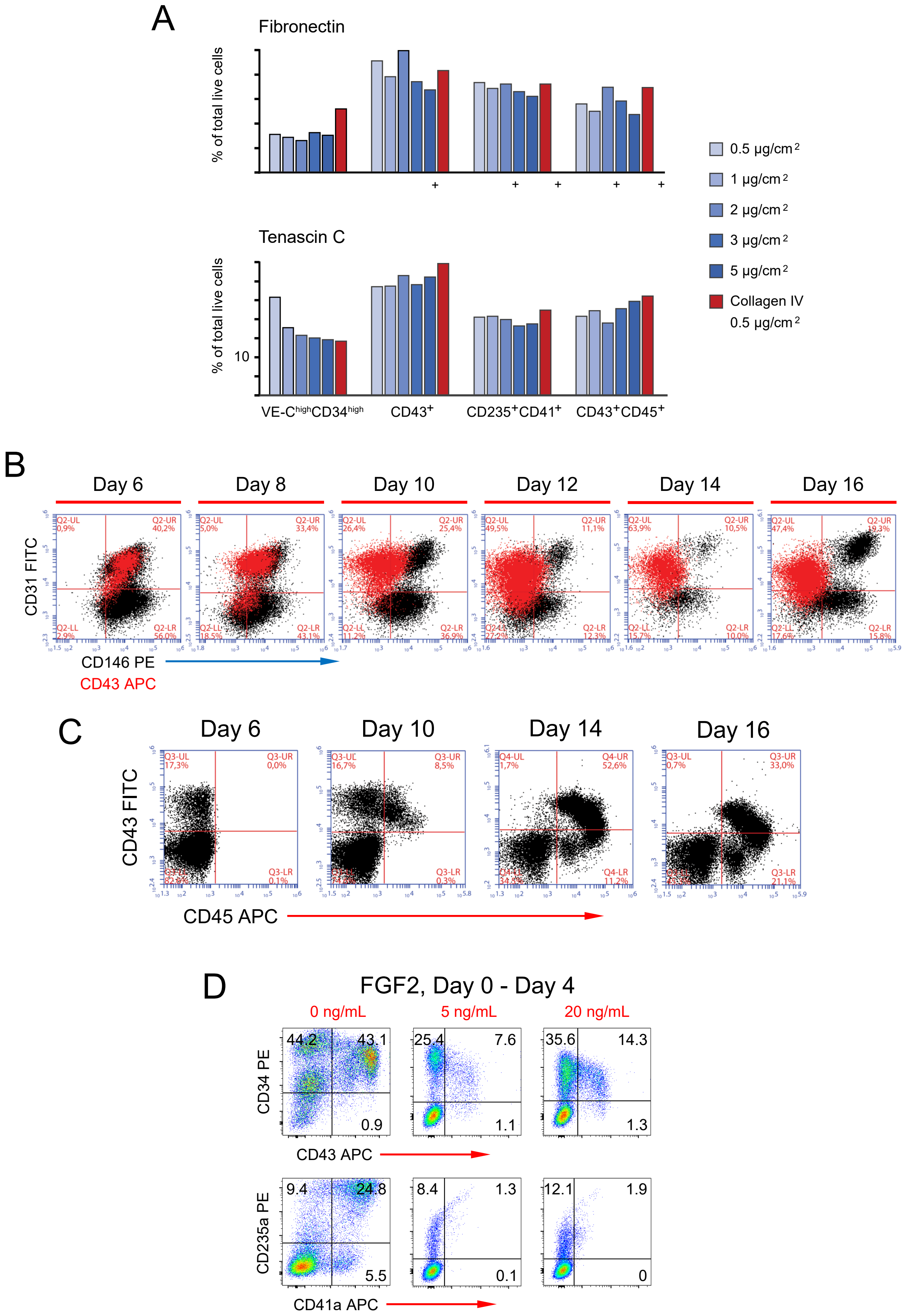
A
Fibronectin
% of total live cells
+
+
+
+
+
Tenascin C
% of total live cells
10
VE-C^high CD34^high
CD43+
CD235+CD41+
CD43+CD45+
0.5 µg/cm2
1 µg/cm2
2 µg/cm2
3 µg/cm2
5 µg/cm2
Collagen IV
0.5 µg/cm2
B
Day 6
Day 8
Day 10
Day 12
Day 14
Day 16
CD31 FITC
CD146 PE
CD43 APC
C
Day 6
Day 10
Day 14
Day 16
CD43 FITC
CD45 APC
D
FGF2, Day 0 - Day 4
0 ng/mL
5 ng/mL
20 ng/mL
44.2
43.1
0.9
25.4
7.6
1.1
35.6
14.3
1.3
CD34 PE
CD43 APC
9.4
24.8
5.5
8.4
1.3
0.1
12.1
1.9
0
CD235a PE
CD41a APC

FIGURE S1

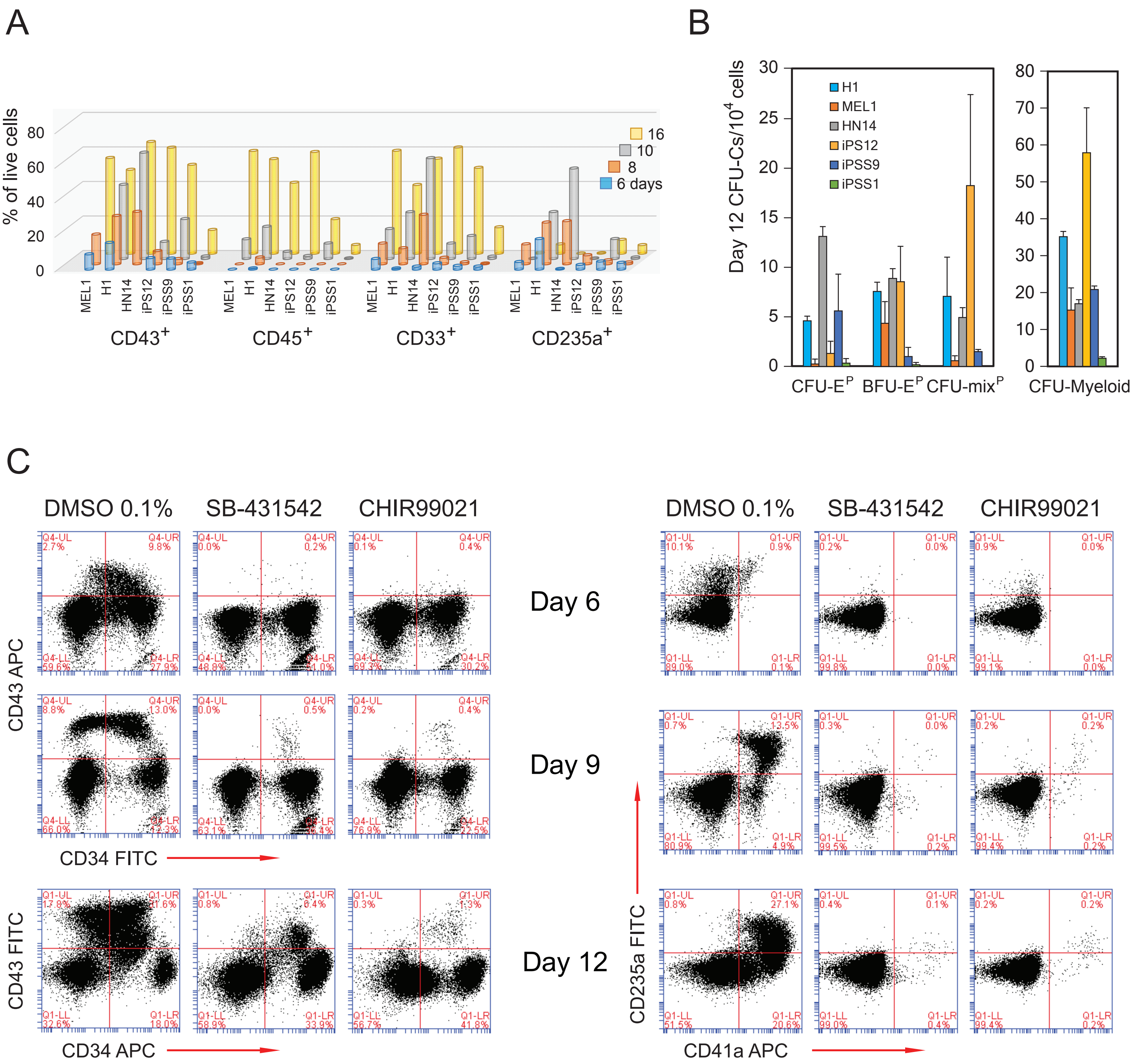
A
% of live cells
16
10
8
6 days
MEL1
H1
HN14
iPS12
iPSS9
iPSS1
CD43+
CD45+
CD33+
CD235a+
B
Day 12 CFU-Cs/10^4 cells
H1
MEL1
HN14
iPS12
iPSS9
iPSS1
CFU-E^P
BFU-E^P
CFU-mix^P
CFU-Myeloid
C
DMSO 0.1%
SB-431542
CHIR99021
Day 6
Day 9
Day 12
CD43 APC
CD34 FITC
CD43 FITC
CD34 APC
CD235a FITC
CD41a APC

FIGURE S2

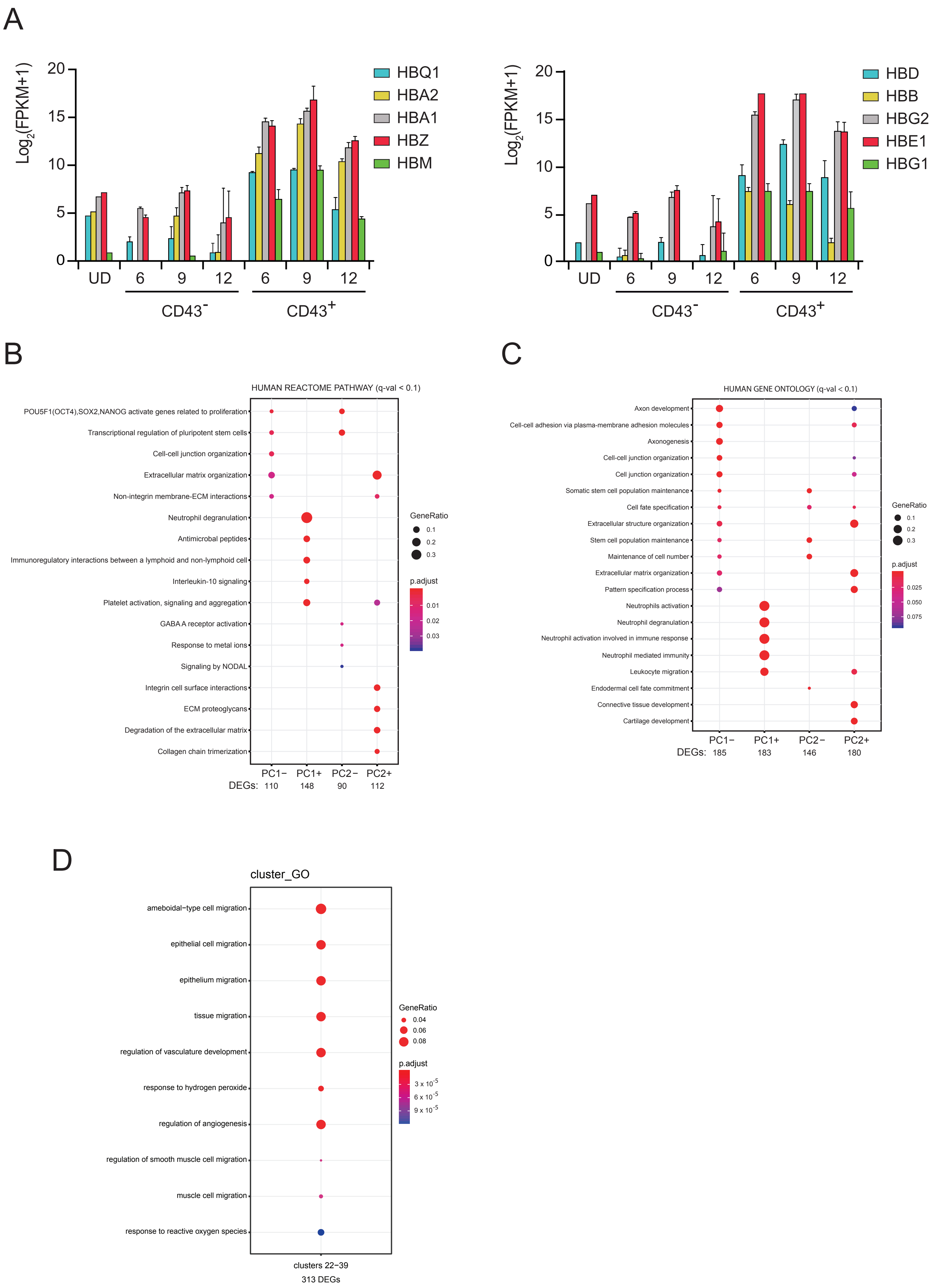
A
Log2(FPKM+1)
HBQ1
HBA2
HBA1
HBZ
HBM
HBD
HBB
HBG2
HBE1
HBG1
UD
6
9
12
CD43-
CD43+
B
HUMAN REACTOME PATHWAY (q-val < 0.1)
POU5F1(OCT4),SOX2,NANOG activate genes related to proliferation
Transcriptional regulation of pluripotent stem cells
Cell-cell junction organization
Extracellular matrix organization
Non-integrin membrane-ECM interactions
Neutrophil degranulation
Antimicrobial peptides
Immunoregulatory interactions between a lymphoid and non-lymphoid cell
Interleukin-10 signaling
Platelet activation, signaling and aggregation
GABA A receptor activation
Response to metal ions
Signaling by NODAL
Integrin cell surface interactions
ECM proteoglycans
Degradation of the extracellular matrix
Collagen chain trimerization
PC1− PC1+ PC2− PC2+
DEGs: 110 148 90 112
GeneRatio
0.1
0.2
0.3
p.adjust
0.01
0.02
0.03
C
HUMAN GENE ONTOLOGY (q-val < 0.1)
Axon development
Cell-cell adhesion via plasma-membrane adhesion molecules
Axonogenesis
Cell-cell junction organization
Cell junction organization
Somatic stem cell population maintenance
Cell fate specification
Extracellular structure organization
Stem cell population maintenance
Maintenance of cell number
Extracellular matrix organization
Pattern specification process
Neutrophils activation
Neutrophil degranulation
Neutrophil activation involved in immune response
Neutrophil mediated immunity
Leukocyte migration
Endodermal cell fate commitment
Connective tissue development
Cartilage development
PC1− PC1+ PC2− PC2+
DEGs: 185 183 146 180
GeneRatio
0.1
0.2
0.3
p.adjust
0.025
0.050
0.075
D
cluster_GO
ameboidal−type cell migration
epithelial cell migration
epithelium migration
tissue migration
regulation of vasculature development
response to hydrogen peroxide
regulation of angiogenesis
regulation of smooth muscle cell migration
muscle cell migration
response to reactive oxygen species
clusters 22−39
313 DEGs
GeneRatio
0.04
0.06
0.08
p.adjust
3 x 10-5
6 x 10-5
9 x 10-5

FIGURE S3

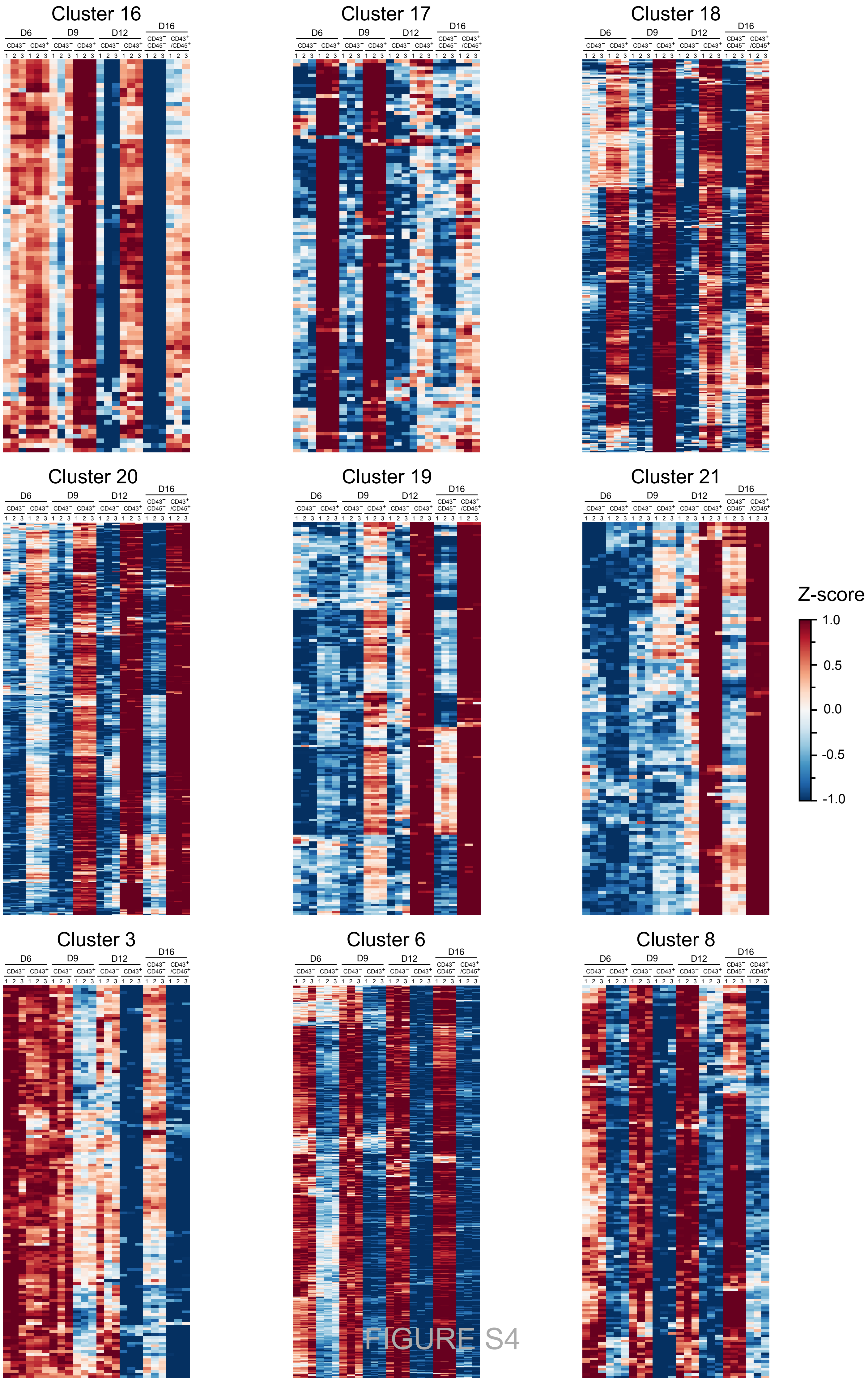
Cluster 16
Cluster 17
Cluster 18
Cluster 20
Cluster 19
Cluster 21
Cluster 3
Cluster 6
Cluster 8
D6
D9
D12
D16
CD43⁻ CD43⁺
CD43⁻ CD45⁻
CD43⁺ /CD45⁺
1 2 3
Z-score
1.0
0.5
0.0
-0.5
-1.0

FIGURE S4

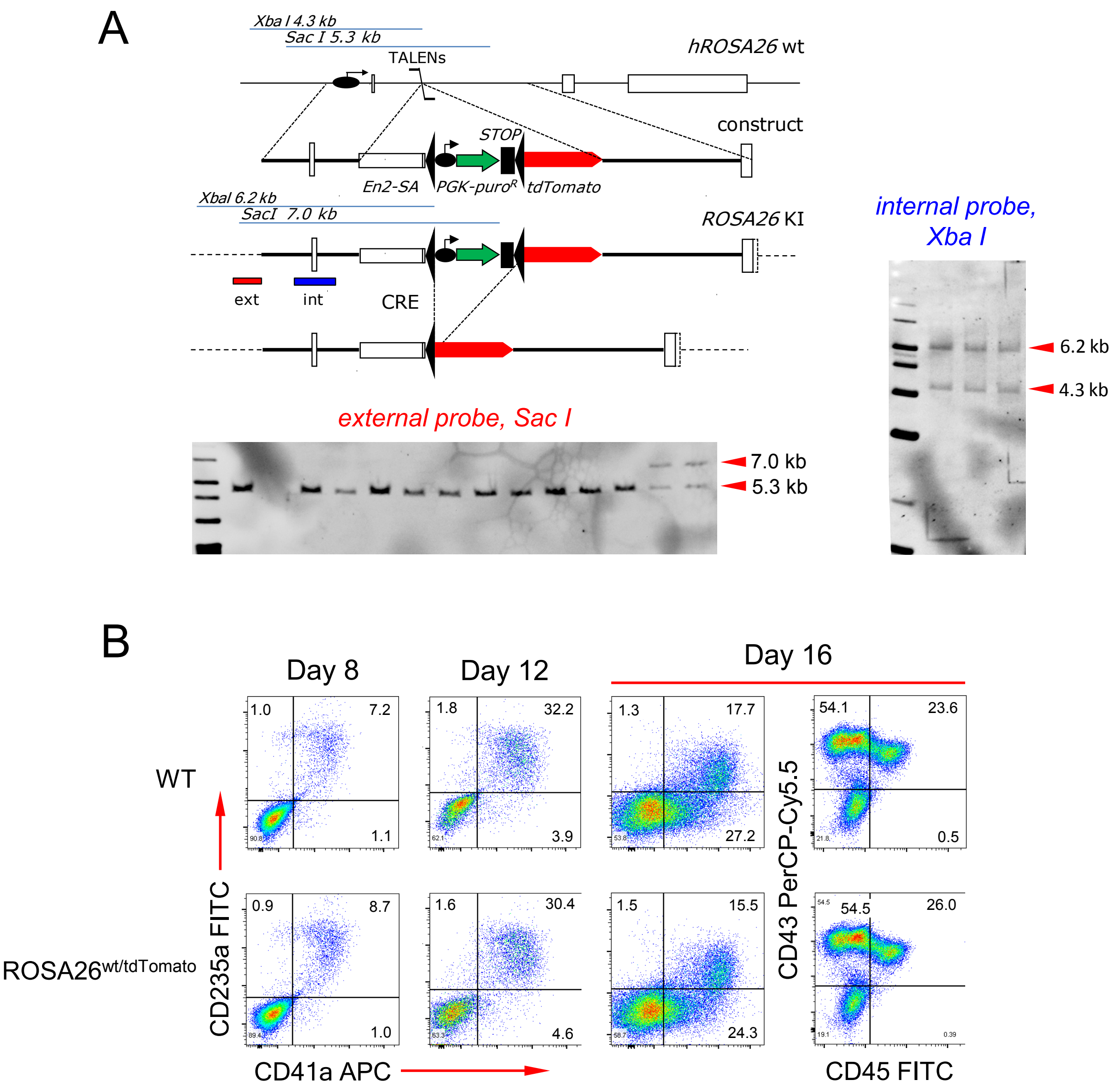
A
Xba I 4.3 kb
Sac I 5.3 kb
TALENs
hROSA26 wt
construct
STOP
En2-SA
PGK-puroR
tdTomato
XbaI 6.2 kb
SacI 7.0 kb
ROSA26 KI
ext
int
CRE
external probe, Sac I
7.0 kb
5.3 kb
internal probe, Xba I
6.2 kb
4.3 kb
B
Day 8
Day 12
Day 16
WT
ROSA26wt/tdTomato
CD235a FITC
CD41a APC
CD43 PerCP-Cy5.5
CD45 FITC
1.0
7.2
1.1
1.8
32.2
3.9
1.3
17.7
27.2
54.1
23.6
0.5
0.9
8.7
1.0
1.6
30.4
4.6
1.5
15.5
24.3
54.5
26.0

FIGURE S5

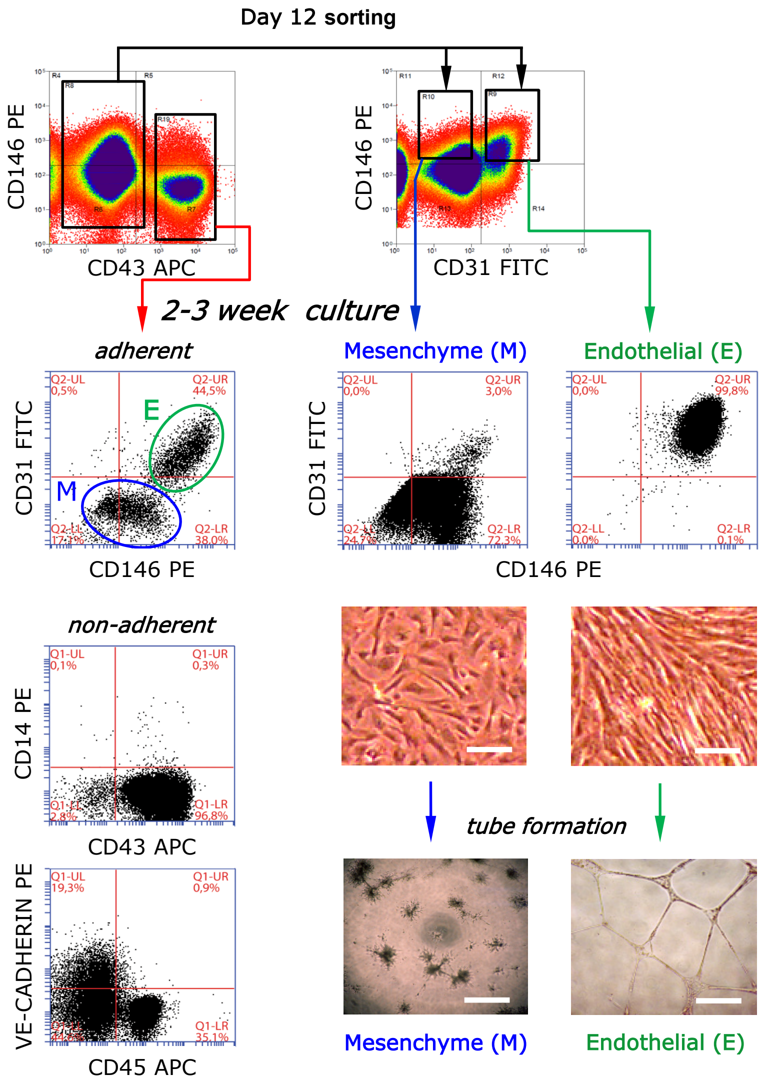
Day 12 sorting
CD146 PE
CD43 APC
CD146 PE
CD31 FITC
2-3 week culture
adherent
Mesenchyme (M)
Endothelial (E)
CD31 FITC
Q2-UL
0,5%
Q2-UR
44,5%
E
M
Q2-LR
38,0%
CD146 PE
CD31 FITC
Q2-UL
0,0%
Q2-UR
3,0%
Q2-LR
72,3%
Q2-UL
0,0%
Q2-UR
99,8%
Q2-LL
0,0%
Q2-LR
0,1%
CD146 PE
non-adherent
CD14 PE
Q1-UL
0,1%
Q1-UR
0,3%
Q1-LL
2,8%
Q1-LR
96,8%
CD43 APC
tube formation
VE-CADHERIN PE
Q1-UL
19,3%
Q1-UR
0,9%
Q1-LR
35,1%
CD45 APC
Mesenchyme (M)
Endothelial (E)

FIGURE S6

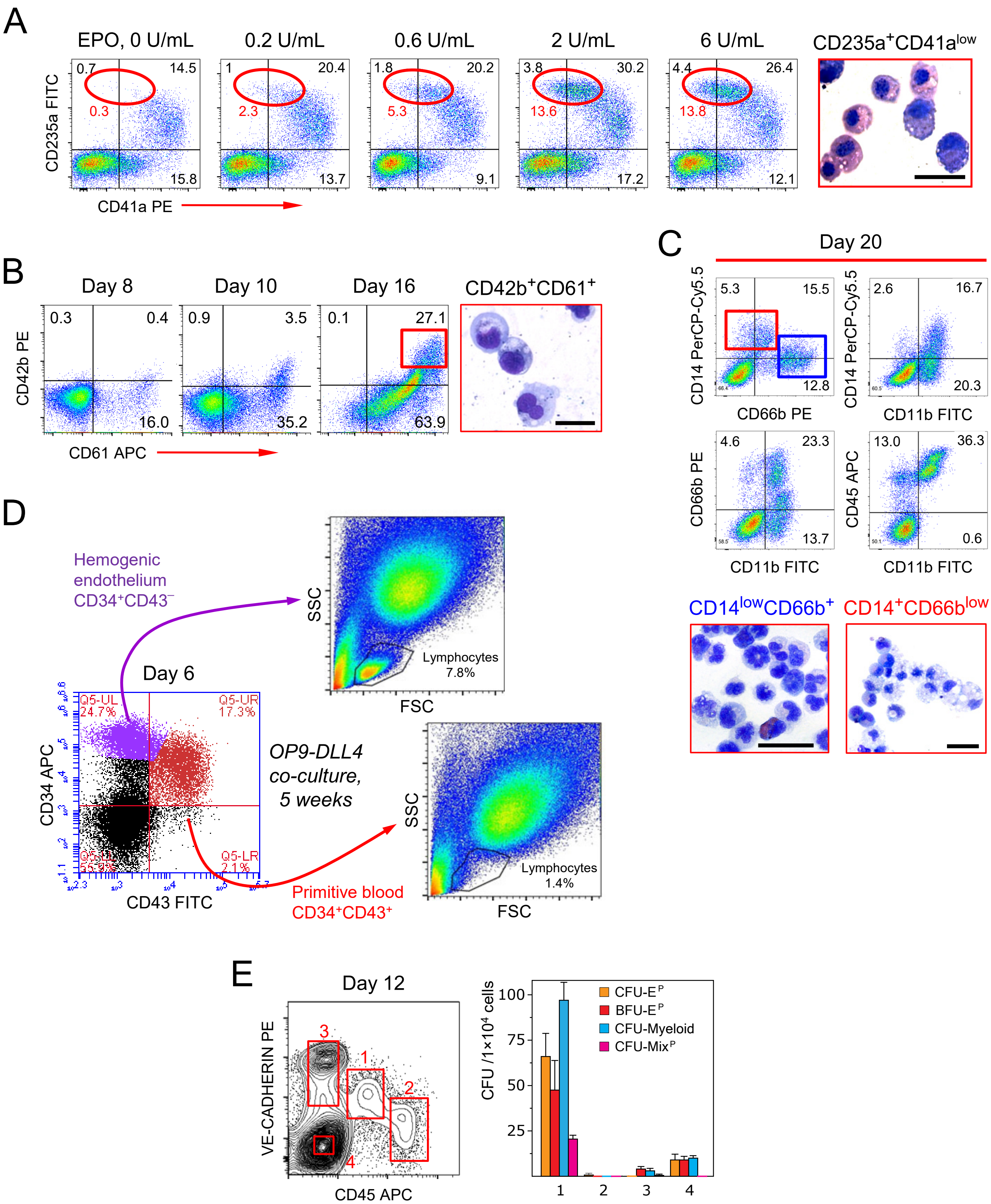
A
EPO, 0 U/mL
0.2 U/mL
0.6 U/mL
2 U/mL
6 U/mL
CD235a+CD41alow
CD235a FITC
CD41a PE
B
Day 8
Day 10
Day 16
CD42b+CD61+
CD42b PE
CD61 APC
C
Day 20
CD14 PerCP-Cy5.5
CD66b PE
CD11b FITC
CD45 APC
CD14lowCD66b+
CD14+CD66blow
D
Hemogenic
endothelium
CD34+CD43−
Day 6
CD34 APC
CD43 FITC
OP9-DLL4
co-culture,
5 weeks
SSC
FSC
Lymphocytes
7.8%
Lymphocytes
1.4%
Primitive blood
CD34+CD43+
E
Day 12
VE-CADHERIN PE
CD45 APC
CFU /1×10⁴ cells
CFU-EP
BFU-EP
CFU-Myeloid
CFU-MixP

FIGURE S7
